# The rhizospheric microbial community structure and diversity of deciduous and evergreen forests in Taihu Lake area, China

**DOI:** 10.1371/journal.pone.0174411

**Published:** 2017-04-05

**Authors:** Zhiwen Wei, Xiaolong Hu, Xunhang Li, Yanzhou Zhang, Leichun Jiang, Jing Li, Zhengbing Guan, Yujie Cai, Xiangru Liao

**Affiliations:** 1 The Key Laboratory of Industrial Biotechnology, Ministry of Education, School of Biotechnology, Jiangnan University, Wuxi, Jiangsu, China; 2 The Key Laboratory of Biotechnology for Medicinal Plant of Jiangsu Province, Jiangsu Normal University, Xuzhou, Jiangsu, China; 3 School of Food and Biological Engineering, Zhengzhou University of Light Industry, Zhengzhou, Henan, China; 4 The Bioscience and Engineering College, Jiangxi Agriculture University, Nanchang, Jiangxi, China; 5 School of Marine and Biological Engineering, Yancheng Teachers University, Yancheng, Jiangsu China; 6 Wuxi Plum Garden and Hengshan Scenic Area, Wuxi, Jiangsu, China; USDA-ARS Salinity Laboratory, UNITED STATES

## Abstract

Soil bacteria are important drivers of biogeochemical cycles and participate in many nutrient transformations in the soil. Meanwhile, bacterial diversity and community composition are related to soil physic-chemical properties and vegetation factors. However, how the soil and vegetation factors affect the diversity and community composition of bacteria is poorly understood, especially for bacteria associated with evergreen and deciduous trees in subtropical forest ecosystems. In the present paper, the microbial communities of rhizospheric soils associated with different types of trees were analyzed by Illumina MiSeq sequencing the V3-V4 region of the 16S rRNA gene. A total of 121,219 effective 16S rRNA gene sequences were obtained, which were classified into 29 bacterial phyla and 2 archaeal phyla. The dominant phyla across all samples (>5% of good-quality sequences in each sample) were *Proteobacteria*, *Acidobacteria*, *Firmicutes* and *Bacteroidetes*. The bacterial community composition and diversity were largely affected by both soil pH and tree species. The soil pH was the key factor influencing bacterial diversity, with lower pH associated with less diverse communities. Meanwhile, the contents of NO_3_^−^ were higher in evergreen tree soils than those associated with deciduous trees, while less NH_4_^+^ than those associated with deciduous trees, leading to a lower pH and indirectly influencing the diversity and composition of the bacteria. The co-occurrence patterns were assessed by network analysis. A total of 415 pairs of significant and robust correlations (co-occurrence and negative) were identified from 89 genera. Sixteen hubs of co-occurrence patterns, mainly under the phyla *Acidobacteria*, *Proteobacteria*, *Firmicutes* and *Bacteroidetes*, may play important roles in sustaining the stability of the rhizospheric microbial communities. In general, our results suggested that local environmental conditions and soil pH were important in shaping the bacterial community of the Taihu Lake zone in east China.

## Introduction

Soil, especially the rhizosphere of plants, is a complex and heterogeneous environment inhabited by various microorganisms. The interactions of plant roots and microorganisms are important for the plant performance as well as ecosystem functioning [1]. It is well known that soil microbes serve as a major reservoir of nutrients for plants through their active involvement in nutrient cycling through organic matter degradation [2], nitrogen cycling and mineral weathering [3, 4], which affect plant growth [5]. Similarly, the functional and taxonomic diversity of rhizospheric microbial communities are strongly influenced by biotic and/or abiotic factors, such as root exudates, competition for nutrients, edaphic characteristics and climate modifications [6–9]. Root exudates are affected by the plant species and genotype [10], including a wide range of compounds, such as amino acids, sugars, enzymes and siderophores [11], which can largely affect the structure of the rhizospheric microbial community and reinforce the activity of microbial communities [5]. While, most of this knowledge has come from cultivation-dependent approaches, yielding only a partial understanding of the interactions among microbes, plants and soil. In fact, cultivation-independent methods for determining microbial communities have suggested that cultivated isolates represent less than 1% of the bacterial taxa [12].

In the last decade, the second generation of high-throughput sequencing (pyrosequencing and Illumina-based surveys) methods have been extensively applied for the analysis of the diversity and structure of the microbial communities in different environments, such as grasslands [13, 14], farmlands [15], forest soils [16–20], arctic seas [21], sea sediments [22–24], hot springs [25] and partial nitritation biofilters [26].

Soil bacteria are also one of the most important components of forest ecosystems [15], and play an essential role in all biogeochemical cycles and nutrient transformations in the soil [27]. Therefore, the study of microbial community structure and diversity in the forest ecosystem is very important.

The Taihu Lake area is approximately 36,900 km^2^ and is located in the Yangtze River Delta of southeast China. It has a subtropical climate with abundant rainfall, and the forest coverage rate of the region surrounding Taihu Lake reaches 42.61% (Environmental protection bureau of Suzhou, 2013). The age structure of the forests in Taihu Lake area mainly belongs to the young forest, accounting for 67–90% [28]. This region has one of the highest population densities and is one of the most developed areas of China [29]. Therefore, the conflicts between humans and environment are inevitable [30], such as the discharge of domestic sewage and industrial waste water, felling of adult trees and application of pesticides, etc. All of these have destroyed the stability of the ecosystem, causing changes in ecosystem structure, diversity and function, and driving the ecosystem into an unstable status. At present, the Taihu Lake area is a pivotal ecologically sensitive zone. The frequent blooms of blue-green algae in northern Taihu Lake are an example. Many studies have focused on the water pollution and restoration in Taihu Lake for a long time [29–34], but few people have noticed the damage to the forest ecosystem, not to mention the microbial community structure and diversity. The forest of the Taihu area is a mixed evergreen and deciduous forest. It is well known that different plant species secrete different root exudates [11]. Therefore, what is the composition of the microbial community in the Taihu Lake area? How do plant species affect the composition and activity of the rhizospheric microbial communities and the soil physic-chemical characteristics? What are the dominant bacterial phyla and their functions in the forest of Taihu Lake Basin? To gain a comprehensive understanding of the ecology and functional potentials, we supposed there were some differences in the rhizospheric microbial communities between evergreen trees and deciduous trees, and evaluated the bacterial diversity and structure of the microbial community of evergreen and deciduous trees by a comparative metagenomic method for understanding the interactions between rhizospheric microorganisms and different types of plants.

The aims of this study were to (1) analyze the traditionally dominant microbial phyla of the forest land in the Taihu Lake area, (2) reveal the correlations among the rhizospheric microbial community, plant species and soil physic-chemical properties in deciduous and evergreen forests, (3) unveil the symbiotic relationships among different microbes in the microbial community.

## Materials and methods

### Ethics statement

All necessary permits for the described field studies were issued by the Administration Bureau of the Mey Blossom Garden, Wuxi, Jiangsu, China.

### Study site and sample collection

The study was conducted at the Mey Blossom Garden (31°55´N, 120°22´E), Wuxi city, adjacent to Taihu Lake, in the Yangtze River delta of China. The forest of the sampling area is approximately 7 km^2^ in size and has a typical subtropical oceanic monsoon climate with a mean annual precipitation of 1048 mm, and a mean growing season length of approximately 220 d. The average temperature at the time of collection was 21–27°C (September 2014). The sampling area vegetation type is representative of typical subtropical forest ecosystems consisting of mixed evergreen and deciduous forest. A total of 204 woody species, including 100 tree species and 104 bush species, from 54 families have been recorded in the study plots (Wuxi Municipal Bureau of Parks and Gardens). The typical deciduous trees include ginkgo (*Ginkgo biloba* L.), crape myrtle (*Lagerstroemia indica* L.), sawtooth oak *(Quercus acutissima* Carruth.), dalbergia (*Dalbergia hupeana* Hance.) and *Wisteria sinensis*. The typical evergreen trees include *Osmanthus fragrans*, *Castanopsis sclerophylla*, camphor tree (*Cinnamomum camphora* (L.) Presl.), *Pinus massoniana*, *Sabina chinensis* (L.) Ant., *Cyclobalanopsis glauca*, *Lithocarpus glaber* and *Castanea henryi*. [28]. The dominant tree species include *Cinnamomum camphora* (L.) Presl., *Sabina chinensis* (L.) Ant., *Ginkgo biloba* L., *Lagerstroemia indica* L., *Osmanthus fragrans* Lour. and *Wisteria sinensis*.

Seven typical native tree species were selected for this study. All of the sampled tree species were more than 20 years old and include the evergreen species *Osmanthus fragrans* Lour. (GH), *Sabina chinensis* (L.) Ant. (HB), *Castanopsis sclerophylla* (Lindl.) Schott. (KC) and *Cinnamomum camphora* (L.) Presl. (ZS), and the deciduous species *Ginkgo biloba* L. (YX), *Lagerstroemia indica* L. (ZW) and *Wisteria sinensis* (ZT). The total abundance of the selected trees are more than 66% in the sampling area [35–36].

Soil sampling was carried out in September 2014. The forest floor litter of the sampling area was removed, and the fibrous roots along the branch roots of the tree were found using a soil knife. The soil samples were collected to a 10 cm depth on the other side of branch roots using an auger with a 10 cm diameter, and the fibrous roots of the tree were carefully selected from the field soil. The non-rhizospheric soil was shaken off and the rhizospheric soil samples were obtained using a brush. Four rhizospheric soil samples from each tree species were collected and mixed into one composite sample. All of the homogenized soil samples were immediately sieved (2 mm mesh) in the field to remove stones and roots, and then transferred to sterile iceboxes, stored at -20°C until molecular analysis. The soil characteristics were determined from the residual soil samples after molecular analysis by referring to the methods of Wu et al. (2012) [37].

### Soil physical and chemical analysis

Soil samples were air-dried and sieved. The pH of the fraction (< 2 mm) was determined by the method of potentiometric analysis in a 1:1 solution (soil to water); Concentrations of total phosphorus and available phosphorus were determined by the chloro-molybdophosphonic blue color method [38] and Olsen and Sommers method [39], respectively. The contents of ammonium and nitrate in the soil samples were measured by the indophenol blue colorimetric method [40] and the phenoldisulfonic acid method [41], respectively. The potassium dichromate-oxidation external heating method was used to determine the soil organic carbon content [42, 43]. The soil texture was measured as described by Will et al. (2010) [14].

### DNA extraction, PCR amplification and sequencing

Approximately 0.2 g (wet weight) of each soil sample in triplicate was used to extract microbial genomic DNA using the E.Z.N.A. Soil DNA kit (Omega Bio-Tek, Doraville, GA, USA) following the manufacturer’s instructions. The DNA integrity was analyzed by electrophoresis in 0.8% (w/v) agarose gel. Then, triplicate DNA extracts of the same soil sample were combined and the concentration of DNA was detected with a Qubit^®^ dsDNA BR Assay kit (Life Technologies Corporation, Carlsbad, CA, USA) according to the manufacturer’s instructions. An Invitrogen Qubit^®^ 2.0 Fluorometer (Life Technologies Corporation, Carlsbad, CA, USA) was used to analyze the concentration of the extracted DNA. The V3-V4 hypervariable region of the bacterial 16S rRNA genes was amplified by the primer pair 341F (CCTACGGGNGGCWGCAG) and 805 R (GACTACHVGGGTATCTAATCC), and a 7-bp unique barcode for each sample was added to the reverse primer to allow multiplex sequencing.

The PCR was performed based on a previous method [44]. The PCR products of different samples were purified by electrophoresis in 2% (w/v) agarose gel and recovered by a SanPrep Column DNA Gel Recovery Kit (Sangon Biotech Shanghai Co., Ltd, Shanghai, China) according to the manufacturer’s instructions, and then quantified according to the previous description with a Qubit^®^ dsDNA BR Assay kit. Subsequently, all of the purified amplicons were pooled at equal molar concentrations for the construction of the PCR amplicon library. The final PCR products were sequenced using a MiSeq benchtop sequencer (Illumina, San Diego, CA, USA) for 250-bp paired-end sequencing at the Sangon Biotech Company in Shanghai.

### Sequence processing

The obtained raw MiSeq-generated gene sequence data were further processed with the software Prinseq (PRINSEQ-lite 0.19.5), including trimming primers, eliminating ambiguous reads and excluding poor quality sequences (average quality score of less than 27), the sequences with fewer than 200 nucleotides were removed from further analysis [45]. The obtained sequence data were corrected using the pre. cluster software package of Mothur 1.30 [46], and the maximum permissible value of mismatch was 1/150 (http://www.mothur.org/wiki/Pre.cluster). Suspected chimeric sequences were removed by the software uchime according to the templates of SILVA database. The sequences similarity thresthod was set at 0.97 for classification into operational taxonomic units (OTUs), and the OTUs were clustered by uclust (http://www.drive5.com/uclust/downloads1_1_579.html). The taxonomic rank of each OTU was analyzed by the Ribosomal Database Project (RDP) Classifier based on Bergey’s taxonomy, and the threshold value of the RDP was 0.8. The alpha diversity including Shannon index and Chao1 index was estimated using the method described by Tao et al. [47]. Unifrac metric was applied to analyze the beta diversity [48].

#### Microarray data accession number

The obtained raw sequences were deposited in the DDBJ database (Accession Number: DRA005560).

### Data and network analysis

To investigate the similarities of different bacterial community composition, principal coordinate analysis (PCoA) was carried out based on the weighted UniFrac distance. Heat map was generated by the gplots package in R version 2.13.0 (R Development Core Team 2006) to compare the top 15 genera in each sample. Redundancy analysis (RDA) was used to estimate the relationships between the environmental factors and 44 abundant genera by the software of Canoco 5.0 (relative abundance ≥ 1% in at least one soil sample) genera, which accounting for 66.32% to 84.08% of all classified genera in each sample.

To visualize the correlations among the bacterial genera in the networks, a correlation matrix was constructed by calculating all possible pairwise Spearman’s rank correlations. Only those genera which with total relative percentage ≥ 0.12% in all samples were taken into consideration. We considered only a Spearman’s correlation coefficient (ρ)>0.6 and statistical significance (*P*<0.01) was a valid co-occurrence event to be a robust correlation, if the Spearman’s correlation coefficient (ρ)<−0.6 and statistical significance (*P*<0.05) was considered a negative event [49, 50]. The statistical analyses were carried out using the Hmisc package in the R environment [50, 51]. In the networks, one node represented one genus, and one edge represented one significant correlation. The network was constructed in the Gephi software [52].

## Results

### General analyses of sequencing data of the rhizospheric soils

In this study, 135,887 raw reads were obtained from the Miseq sequencing analysis of 7 soil samples. After trimming, denoising and removing chimeras, 121,219 effective sequences remained with an average length of 450 bp (Table 1). Then, sequences were clustered by 3% dissimilarity, we identified 14,124 bacterial OTUs. All of the effective sequences were classified into 29 bacterial groups and 2 archaeal groups. The highest number of sequences (23,340 sequences) was in the ZT sample, and the lowest number of sequences (7,310 sequences) was in the YX sample. Therefore, the alpha and beta diversity analyses were calculated by normalizing to 7,310 sequences in the other libraries. All classified sequences were assigned to the bacterial or archaeal domain, but only 0.07–0.20% of the sequences belonged to the archaeal domain, so this was a rare group.

**Table 1:** Bacterial richness indices of the 7 samples in this study.

Samples	Number of effective sequences	Number of OTUs^a^	Coverage (%)	Chao1^a^	Shannon index^a^
ZT	23,340	2597.60	87.71	5993.66	6.88
YX	7,310	2486.00	79.11	5660.66	6.83
ZW	16,648	2434.56	85.49	5837.12	6.69
ZS	16,789	2022.24	88.05	4907.14	6.25
KC	24,687	1762.64	90.64	4641.77	5.65
HB	14,532	1616.80	90.26	3715.13	5.59
GH	17,913	1434.48	91.13	3669.13	5.29

ZW: *Lagerstroemia indica* L., HB: *Sabina chinensis* (L.) Ant., KC: *Castanopsis sclerophylla* (Lindl.) Schott., ZS: *Cinnamomum camphora* (L.) Presl., GH: *Osmanthus fragrans* Lour., ZT: *Wisteria sinensis*, YX: *Ginkgo biloba* L.

^a^ Indices (OTUs, Chao1 and Shannon) were calculated based on the randomly selected 7,310 sequences. Cutoff = 0.03

#### α-diversity

The coverage of seven samples was from 79.11% to 91.13% (Table 1). The OTUs obtained from each sample were shown in the rarefaction curves (S1 Fig). According to the number of OTUs, the highest richness was in the ZT sample, followed by the YX and ZW samples, and the GH sample had the lowest richness. The Shannon index showed a similar pattern to that of the OTUs, but the Chao1 index differed from the Shannon index and the number of OTUs. The Shannon index of YX was higher than that of ZW, while the Chao1 index of YX was lower than that of ZW, which demonstrated the sum of bacterial species in the soil sample of ZW was higher than that in the sample of YX.

#### β-diversity

**Fig 1:**
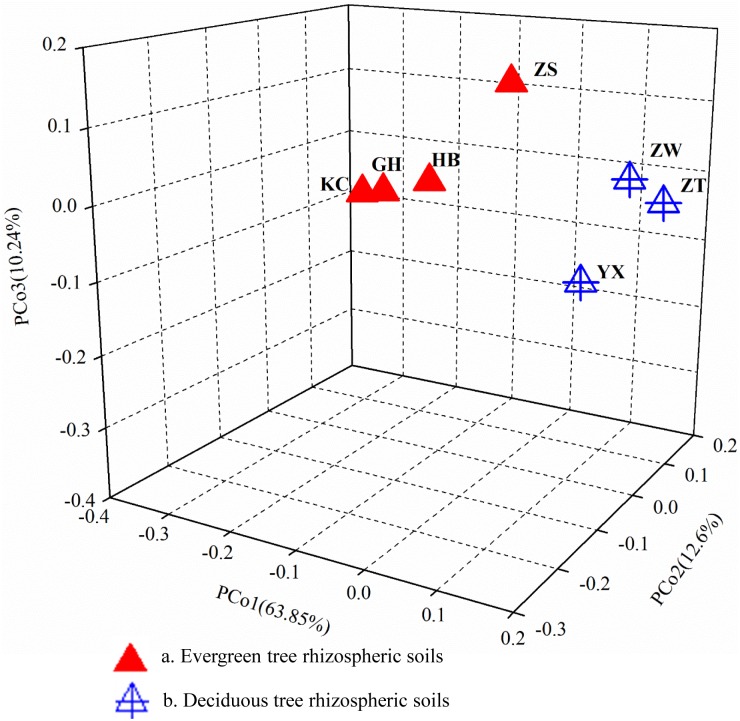
PCoA plot based on the weighted UniFrac distance.

All the soil samples were clustered into two groups, evergreen trees and deciduous trees (Fig 1). The type (evergreen and/or deciduous) of trees was apparently one of the influencing factors (without considering the geochemical factors) in the first principal-coordinate axis (PCo1), and contributed 63.85% of the total variation, which suggested that the microbial community composition was frequently related to the type of trees. For example, *Acidobacteria* and *Firmicutes* associated with evergreen trees were more abundant than deciduous trees, while *Proteobacteria* showed the opposite pattern (S2 Fig).

### Bacterial community composition in each soil sample

**Fig 2:**
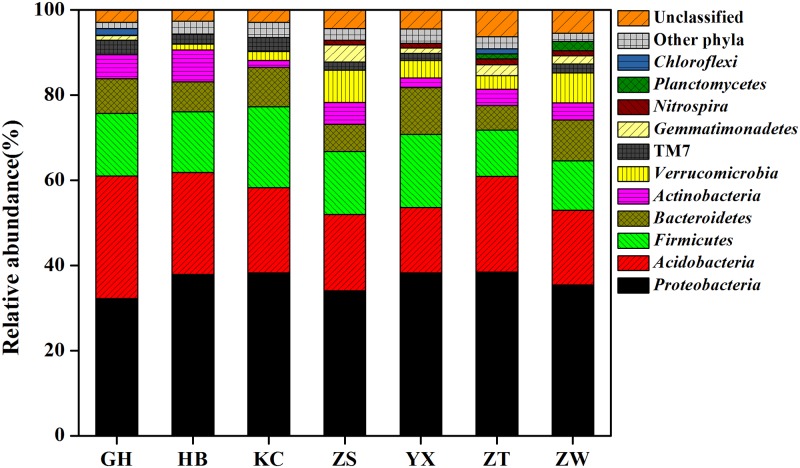
Phylum-level bacterial sequence diversity from each rhizospheric soil sample. The taxa represented accounted for >1% abundance in at least one sample. Other phyla had a maximum abundance of <1% in any sample.

At the phylum level, the classification results for each soil sample are depicted in Fig 2. *Proteobacteria*, *Acidobacteria*, *Firmicutes* and *Bacteroidetes* were the dominant phyla (>5% of good-quality sequences in each sample), accounting for 32.23–38.45, 15.4–28.78, 10.92–19.03, 5.75–10.98% of the total good-quality sequences, respectively. Other subdominant phyla (>1% of good-quality sequences in at least one sample) included *Actinobacteria*, *Verrucomicrobia*, TM7, *Gemmatimonadetes*, *Nitrospira*, *Planctomycetes* and *Chloroflexi*, accounting for 1.65–7.47, 0.39–7.61, 0.82–3.42, 0.72–4.06, 0.06–1.36, 0.31–2.20 and 0.25–1.66% of the total sequences in each soil sample, respectively (Fig 2; S1 Table). The 11 above-mentioned phyla accounted for 91.72–96.58% in each sample. For the other twenty phyla, none of the effective sequences occurred at ˃1% abundance of the good-quality sequences in at least one soil sample. Thus, these phyla were defined as rare phyla, including *Chlamydiae*, *Fusobacteria*, *Euryarchaeota*, OD1, *Elusimicrobia*, *Armatimonadetes*, WS3, *Chlorobi*, *Spirochaetes*, *Fibrobacteres*, *Cyanobacteria*/*Chloroplast*, *Tenericutes*, BRC1, SR1, *Deinococcus*-*Thermus*, *Synergistetes*, *Crenarchaeota*, OP11, *Aquificae* and *Deferribacteres*. Additionally, the phyla *Euryarchaeota* and *Crenarchaeota* belonged to the archaea domain, accounting for 0.05–0.19% and 0–0.02% of the total sequences in each soil sample, respectively (S1 Table).

### Similarity and differences in bacterial community structure between different tree types

At the class level, a total of 68 bacterial classes were identified in the seven rhizospheric soil samples (S2 Table). A total of 40 classes were shared by all seven soil samples, accounting for 86.48–92.71% of the total good-quality sequences in each sample. The predominant classes in evergreen tree soils (>5% of the good-quality sequences in each evergreen rhizospheric soil sample) were *Gammaproteobacteria*, *Clostridia*, *Acidobacteria*_Gp2, *Acidobacteria*_Gp1 and *Alphaproteobacteria*, accounting for 48.16–65.81%, while in the deciduous tree soils, the dominant classes were *Gammaproteobacteria*, followed by *Alphaproteobacteria*, *Clostridia*, *Acidobacteria*_Gp6 and *Betaproteobacteria*, accounting for 44.69–52.27% (S3 Table). The classes *Gammaproteobacteria*, *Clostridia* and *Alphaproteobacteria* were shared by evergreen tree soils and deciduous tree soils as dominant classes.

We found the relative abundances of *Gammaproteobacteria*, *Acidobacteria*_Gp1, *Acidobacteria*_Gp2 and *Clostridia* were higher in evergreen tree soils than that in deciduous tree soils in general, whereas *Alphaproteobacteria*, *Betaproteobacteria*, *Deltaproteobacteria* and *Acidobacteria*_Gp6 showed the opposite pattern (S3 Table). Meanwhile, some differences between evergreen and deciduous tree soils were discovered within individual lineages. The members of *Acidobacteria* were dominant in all soil samples, accounting for approximately 20% of all good-quality sequences. We found 16 and 20 of the subgroups in evergreen soils and deciduous tree soils, respectively. The subgroups Gp1 and Gp2 were abundant (>1% of effective sequences in each soil sample) in evergreen soils (low pH), and Gp4, Gp5 and Gp6 were relatively abundant (>1% of effective sequences in each soil sample) in deciduous tree soils (high pH). The subgroup Gp3 was distributed in the two types of rhizospheric soils, but it was higher in the low pH conditions. Furthermore, Gp9, Gp18, Gp20 and Gp22 were only shared by deciduous tree soils (high pH; S3 Table). In the other lineage of *Proteobacteria*, the class *Gammaproteobacteria* was abundant at low pH, *Alphaproteobacteria* predominated at intermediate pH, *Betaproteobacteria* and *Deltaproteobacteria* were abundant in higher pH soils.

**Fig 3:**
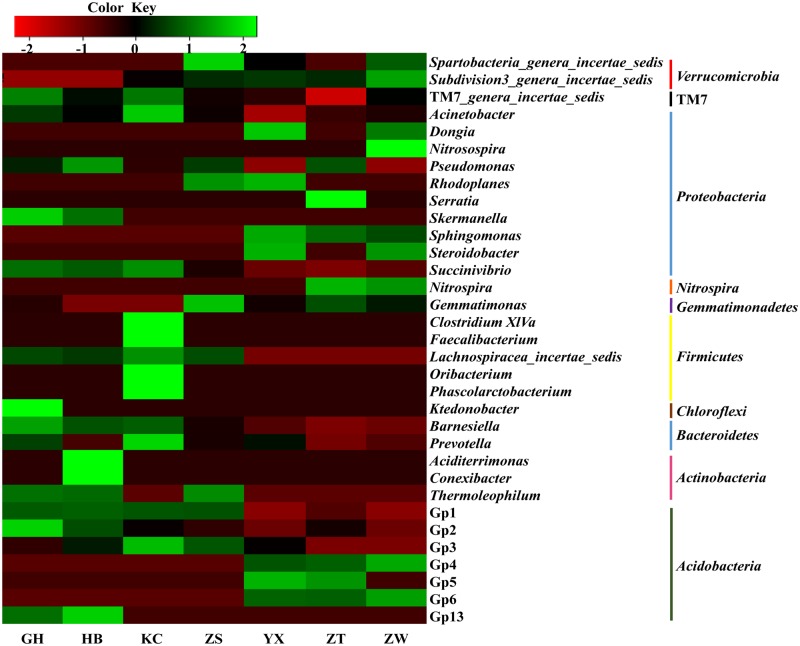
Heatmap of top 15 genera in each rhizospheric soil sample. The color intensity in each box indicates the relative percentage of a genus in each sample.

At the genus level, a total of 737 classified genera were obtained from all soil samples, and 171 genera were shared by all the 7 soil samples, accounting for 81.81–95.49% of the classified sequences in each sample (S4 Table). One hundred and eighty-nine genera were observed in only one sample, accounting for <0.6% of the classified sequences in each sample (S5 Table). The top 15 classified genera in each soil sample were selected (a total of 33 genera for all 7 samples), and their abundances were compared to those in other soil samples by heatmap analysis (Fig 3). Twelve genera (dominant genera) were abundant (>1% of the classified sequences) in at least 5 soil samples (S6 Table), accounting for 26.72–66.58% in each soil sample, including *Succinivibrio*, Gp2, Gp1, Gp3, TM7*_genera_incertae_sedis*, *Barnesiella*, *Acinetobacter*, *Pseudomonas*, *Prevotella*, *Lachnospiracea_incertae_sedis*, *Gemmatimonas* and *Subdivision3_genera_incertae_sedis*. While, the dominant genera were significantly different between the evergreen group and deciduous group. The dominant genera of the evergreen group (>1% of the classified sequences in each evergreen tree rhizospheric soil sample) included *Succinivibrio*, Gp2, Gp1, TM7*_genera_incertae_sedis*, *Barnesiella*, *Acinetobacter*, *Pseudomonas*, *Prevotella*, *Lachnospiracea_incertae_sedis*, Gp3 and *Phascolarctobacterium*, accounting for 42.89–66.10% (S7 Table). The genera *Succinivibrio*, *Barnesiella*, *Acinetobacter*, *Prevotella*, *Gemmatimonas*, Gp3, *Subdivision3_genera_incertae_sedis*, *Steroidobacter*, *Sphingomonas*, Gp6, *Nitrospira*, *Dongia*, Gp4, *Spartobacteria_genera_incertae_sedis* and Gp5 were dominant (>1% of the classified sequences in each deciduous tree rhizospheric soil sample) in the deciduous group, accounting for 40.84–51.12% (S8 Table). The most abundant genus was *Succinivibrio* in all soil samples, accounting for 13.97–22.92% of the classified genera in the evergreen tree soil samples and 9.30–11.43% of the classified genera in the deciduous tree soil samples. While the dominant genera affiliated with *Acidobacteria* were significantly different, the genera Gp1, Gp2 and Gp3 were dominant in evergreen tree soil samples (S7 Table), conversely, Gp4, Gp5 and Gp6 were the dominant genera in deciduous tree soil samples in addition to Gp3 (S8 Table).

### Network analysis

**Fig 4:**
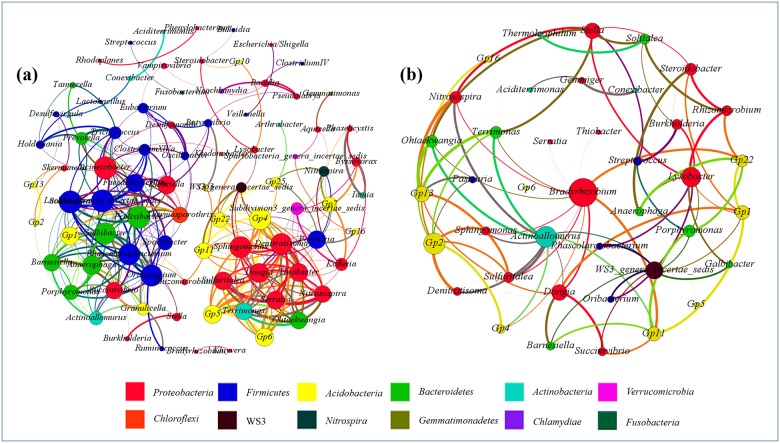
Networks of co-occurring microbial genera in rhizospheric samples based on correlation analysis. A connection indicates a statistically significant (*P*˂0.01) strongly positive correlation (a) (Spearman’s ρ>0.6) or a negative correlation (b) (Spearman’s ρ˂−0.6). For each panel, the size of each node is proportional to the number of connections, the nodes of the same color were affiliated with the same phylum, and the thickness of each connection between two nodes is proportional to the value of Spearman’s correlation coefficients of >0.6 or ˂−0.6.

The co-occurrence patterns were evaluated by network analysis based on the hypothesis that bacterial assembly in rhizospheric soil samples was not random. The bacterial co-occurrence patterns of soil samples were analyzed based on robust and significant correlations (positive correlation *P*<0.01; negative correlation *P*<0.05), and 86 bacterial genera (nodes) and 330 pairs of robust and significant correlations (edges) were identified in the positive correlative network (Fig 4a). There were 16 hubs (highly connected genera ≥8 edges per node) in the co-occurrence pattern, including *Actinoallomurus*, *Barnesiella*, *Dongia*, Gp4, *Lachnospiracea_incertae_sedis*, Gp11, *Acinetobacter*, *Anaerophaga*, *Denitratisoma*, *Galbibacter*, *Faecalibacterium*, Gp1, Gp5, Gp22, Gp6 and *Nitrosospira*, and 6 of them belonged to *Acidobacteria*, 4 of them belonged to *Proteobacteria*, and the other genera were under the phyla *Firmicutes*, *Bacteroidetes* and *Actinobacteria*. The results showed that the phyla *Acidobacteria* and *Proteobacteria* played important roles in sustaining the stability of the rhizospheric microbial communities.

Several topological properties were calculated and described the complex pattern of correlations among the microbial genera in the network of rhizospheric soil samples [53]. The average network distance was 2.879 edges between all pairs of nodes (average path length), and the diameter (the longest distance) was 11 edges for the positive network. In addition, the modularity index was 0.551 (>0.4), which indicated the positive network possessed a modular structure [54].

The genera of different phyla possessed a high co-occurrence incidence (73.03%) based on structural analysis. The phyla *Proteobacteria*, *Firmicutes*, *Acidobacteria* and *Bacteroidetes* showed robust correlations, accounting for 52.42% (of a total of 73.03%). Among any two different phyla, the co-occurrence incidence of *Proteobacteria* and *Acidobacteria* was the highest, up to 15.45%. The co-occurrence incidence of the same phylum (intraphylum) was 26.97%, meanwhile, the maximal co-occurrence incidence (10.30%) was found in the genera belonging to *Proteobacteria*, followed by *Firmicutes* (8.18%) and *Acidobacteria* (4.55%). Furthermore, a total of 85 pairs of significant and robust negative correlations were identified from 40 genera (Fig 4b). Most of the genera belong to *Proteobacteria*, *Acidobacteria* and *Bacteroidetes* (31 hubs). *Bradyrhizobium*, *Actinoallomurus* and Gp2 were the main hubs (≥7 edges).

### Relationships between microbial communities and environmental variables

Among all the biogeochemical attributes, we found the values of pH increased gradually from GH to ZW (Table 2), meanwhile, the pH level of the evergreen tree group was lower than that of the deciduous tree group. The contents of NH_4_^+^ and AP followed the same trend as pH, while the contents of NO_3_^−^ changed conversely to pH. The levels of TP, SOC and soil texture did not show obvious trends. The results indicated that different plant species favored different nitrogen resources, which consequently increased or decreased the pH of rhizospheric soils.

**Table 2:** Biogeochemical indices and soil texture of rhizospheric soil samples.

Variables	Value for indicated tree species (mean ± SD)						
	GH	HB	KC	ZS	YX	ZT	ZW
SOC (g/kg)	26.37±0.61	25.42±1.55	28.72±3.11	29.79±0.15	9.53±0.68	25.74±0.72	31.06±0.20
pH	3.84±0.17	4.19±0.02	4.59±0.03	5.25±0.07	6.96±0.05	7.67±0.11	7.79±0.02
AP (mg/kg)	0.41±0.01	0.35±0.03	0.34±0.03	0.33±0.02	0.23±0.01	0.18±0.02	0.16±0.02
TP (mg/kg)	36.01±1.64	26.48±0.59	23.40±0.80	23.10±1.28	23.61±1.56	12.98±0.61	14.87±0.99
NO_3_^−^ (mg/kg)	9.43±0.43	9.32±0.19	8.88±0.21	7.06±0.51	3.42±0.19	3.19±0.13	0.76±0.06
NH_4_^+^ (mg/kg)	3.93±0.06	4.20±0.14	5.26±0.13	6.33±0.22	7.37±0.30	10.54±0.04	10.77±0.21
Sand (g/kg)	56.56±7.97	50.69±12.39	64.52±5.43	52.51±4.23	63.89±7.78	46.92±7.35	54.19±9.08
Silt (g/kg)	554.78±67.21	527.48±44.12	526.20±63.30	565.69±75.00	545.07±51.93	550.93±37.08	519.80±17.76
Clay (g/kg)	388.66±75.07	421.83±54.37	409.28±65.75	381.80±74.75	391.04±48.84	402.15±44.39	426.01±36.11

**Fig 5:**
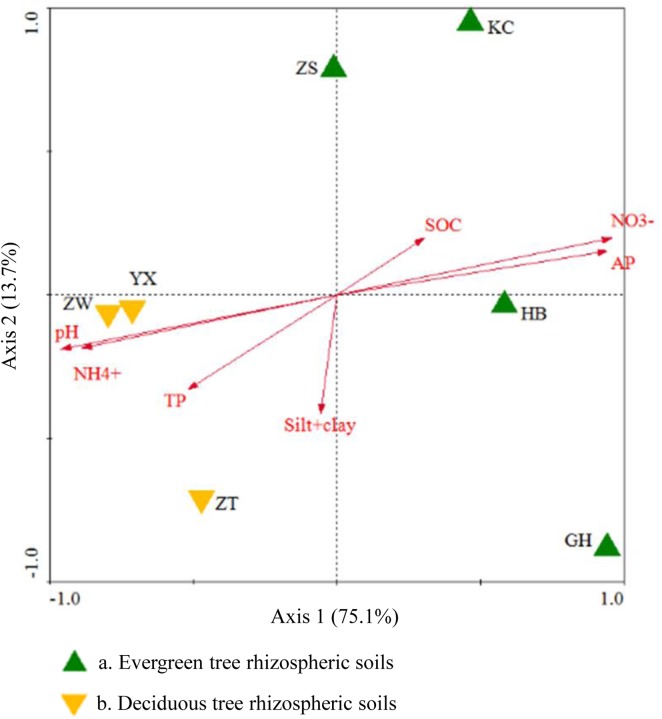
Redundancy analysis of biogeochemical attributes, soil texture and soil samples. Arrows indicate the direction and magnitude of biogeochemical attributes associated with microbial community structures.

**Fig 6:**
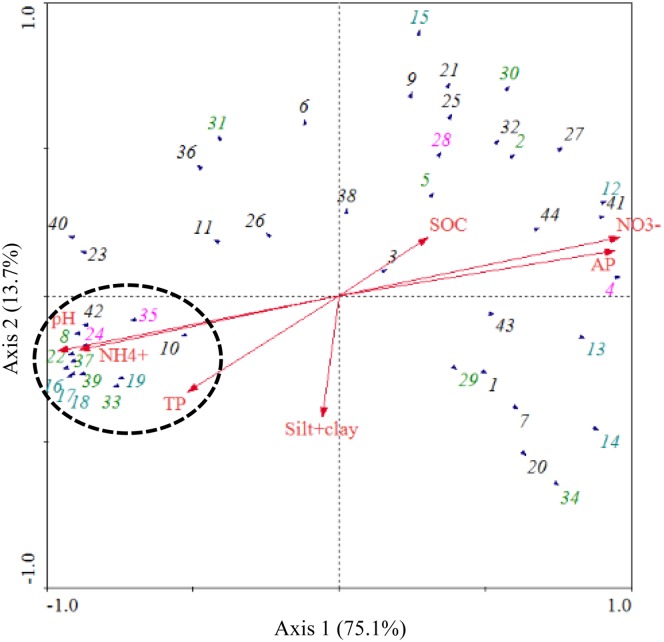
Redundancy analysis of biogeochemical attributes, soil texture and 44 abundant genera in all samples. The relative abundance of 44 abundant genera > 1% in at least one sample. Arrows indicate the direction and magnitude of biogeochemical attributes associated with microbial community structures. 1: Aciditerrimonas, 2: *Acinetobacter*, 3: Bacillus, 4: *Barnesiella*, 5: *Burkholderia*, 6: Clostridium XlVa, 7: Conexibacter, 8: *Dongia*, 9: Faecalibacterium, 10: Gemmata, 11: Gemmatimonas, 12: Gp1, 13: Gp13, 14: Gp2, 15: Gp3, 16: Gp4, 17: Gp5, 18: Gp6, 19: Gp7, 20: Ktedonobacter, 21: Lachnospiracea_incertae_sedis, 22: Nitrosospira, 23: Nitrospira, 24: *Ohtaekwangia*, 25: Oribacterium, 26: Oscillibacter, 27: Phascolarctobacterium, 28: *Prevotella*, 29: *Pseudomonas*, 30: *Rhizomicrobium*, 31: *Rhodoplanes*, 32: Ruminococcus, 33: *Serratia*, 34: *Skermanella*, 35: *Solitalea*, 36: Spartobacteria_genera_incertae_sedis, 37: *Sphingomonas*, 38: Sporobacter, 39: *Steroidobacter*, 40: Subdivision3_genera_incertae_sedis, 41: Succinivibrio, 42: Terrimonas, 43: Thermoleophilum, 44: TM7_genera_incertae_sedis. According to the distribution of the genera *Bacteroidetes* (purple font), *Acidobacteria* (blue font), and *Proteobacteria* (green font) observed in Fig 6, one circle was manually drawn.

Shannon’s diversity index is widely used in ecology [55, 56]. It was significantly correlated with the pH of soil samples (*R*^2^ = 0.9216, *P*<0.01; see S3 Fig). Redundancy analysis was conducted using 44 abundant genera (relative abundance >1% in at least one soil sample; accounting for 66.32% to 84.08%) and environmental variables (Figs 5 and 6). In general, the first two axes explained 88.8% of the variation in microbial composition, and the species-environment correlations of both axes were higher than 95% (pseudo-canonical correlation), which suggested a significant correlation between environmental factors and microbial community structure. Four explanatory variables (NO_3_^−^, pH, AP and NH_4_^+^) had significant contributions (*P*˂0.05) decreasing from 68.4 to 56.1% by Pearson’s test. The variants of NO_3_^−^ and AP had strongly positive correlations with bacterial communities in all evergreen tree rhizospheric soils, while pH, NH_4_^+^ and approximately 13 genera belonged to the phyla *Proteobacteria*, *Acidobacteria* and *Bacteroidetes* positively correlated with microbial communities in the deciduous tree soils (Figs 5 and 6).

## Discussion

### Impact of plant species on the relative abundances of bacterial taxa

Many environmental factors, such as soil organic matter chemistry [57], plant species [58], soil pH [59] and environmental factors [60] can influence soil microbial diversity. For example, different root exudate compositions from different plant species can select distinct microbial populations in the rhizosphere [61, 62]. In our study, the type of trees was found to be one of the influencing factors. Seven soil samples were clustered into an evergreen tree group and a deciduous tree group (Fig 1), which suggested that the microbial community composition was frequently related to the type of trees. For example, the Chao1 index of YX was lower than that of ZW, indicating the microbial biomass depends on the quantity of soil organic carbon [63], because the content of SOC was 9.53 g kg^-1^, which is lower than any others (Table 2). *Ginkgo biloba* has a long history of use in traditional Chinese medicine, and is used to produce many products, including diterpene ginkgolides, bilobalide, ginkgolic acid, ginkgo flavonol glycosides and tannic acid [64]. Some studies reported the products extracted from *G*. *biloba* (including leaves, bark, ginkgo nuts and roots) could inhibit or kill many kinds of microorganisms [65, 66]. Therefore, the exudates of *G*. *biloba* roots could inhibit the growth of bacteria near the roots, leading to the low sequences detected. Meanwhile, soil microbes also serve as a major reservoir of organic carbon [67], and therefore the low abundance of microorganisms resulted in the low content of soil organic carbon. However, soil pH as the major determining factor of soil bacterial communities has been documented by many studies. Griffiths et al. (2011) observed the determining effect of pH on bacterial taxa in British soils [68]. Chu et al. (2010) also reported the composition and diversity of arctic soil bacterial communities were structured depending on local variation in soil pH rather than geographical proximity [59]. Therefore, the shifts in the composition of the bacterial community connected with a change from evergreen trees to deciduous trees could be due to different plant types favoring different nutrients and/or producing different root exudates, which structured the composition and diversity of the bacterial communities by indirectly changing the physic-chemical characteristics of the rhizospheric soils [58].

### Analysis of similarity and differences in bacterial community composition

The dominant bacterial phyla (*Proteobacteria*, *Acidobacteria*, *Firmicutes* and *Bacteroidetes*) identified in this study were not significantly different from those in most other soils. Sul et al. (2013) observed that 73–86% of the total sequences from tropical agricultural land were assigned to *Acidobacteria*, *Proteobacteria*, *Firmicutes*, *Actinobacteria*, *Verrucomicrobia*, *Gemmatimonadetes* and *Bacteroidetes* [69]. Chu et al. (2010) found the dominant phyla were *Proteobacteria*, *Acidobacteria*, *Actinobacteria* and *Bacteroidetes* in arctic soils [59]. Nacke et al. (2011) found that *Proteobacteria*, *Acidobacteria*, *Actinobacteria* and *Betaproteobacteria* were the dominant taxa in German forest soils [27]. All of these suggested that dispersal limitation was less important in determining bacterial community [70, 71].

The members of *Acidobacteria* were dominant in all soil samples, accounting for approximately 20% of all good-quality sequences, which was consistent with many reports [27, 72]. *Acidobacteria* are widely distributed across a range of ecosystems, particularly in soils [73–75], thus, the members of *Acidobacteria* must play an important role in ecosystem. However, the number of cultivated and classified acidobacterial strains remains low [76], let alone understand the physiology and metabolic functions. The phylum *Acidobacteria* includes 26 subgroups [76], of which Gp1, Gp2, Gp3, Gp4 and Gp6 are the most abundant in various soils [74, 77]. In our study, the subgroups Gp1 and Gp2 were abundant in evergreen soils (low pH), and Gp4, Gp5 and Gp6 were relatively abundant in deciduous tree soils (high pH). The subgroup Gp3 was distributed in the two types of rhizospheric soils, but was more abundant under the low pH conditions. The results corresponded roughly with some previous reports. Chu et al. (2010) found the groups 1–3 of *Acidobacteria* decreased when soil pH increased, while Gp4 and Gp6 showed an opposite pattern [59]. Furthermore, Griffiths et al. (2011) also reported that low pH soils were dominated by Gp1 and that Gp6 notably increased in higher pH soils [68]. In the other lineage of *Proteobacteria*, the class *Gammaproteobacteria* was abundant at low pH, *Alphaproteobacteria* predominated at intermediate pH, and *Betaproteobacteria* and *Deltaproteobacteria* were abundant in higher pH soils. Our results corresponded to the study of Griffiths et al. (2011), in which *Alphaproteobacteria* was dominant at low pH, whereas *Betaproteobacteria* increased with pH [68]. All of these suggested that soil pH was the fundamental factor that strongly influenced the structure of the soil bacterial community (Table 2 and S3 Table). However, the tree type could directly affect the physic-chemical properties, which then influences the bacterial community structure of rhizospheric soils.

One hundred and seventy-one genera were shared by all 7 soil samples, which accounted for 81.81–95.49% of the classified sequences in each sample. However, 189 genera were observed in only one sample. There might be many factors that affected the appearance of unique genera in the different soil samples, such as plant excreta, pH and other nutritional components. Of all the classified genera, the most abundant was *Succinivibrio*. Little is known about the role of *Succinivibrio*, which was determined to be more abundant in the gut of bees and the rumens of cattle when fed a starch rich diet [78, 79], as well as in the feces of horses on a high starch diet [80]. The enrichment of *Succinivibrio* in the rhizospheric soil samples could suggest that starch metabolism occurred in the soils. Many studies have reported that *Acidobacteria* are abundant in association with lower pH values [81, 82], but we discovered the opposite pattern for the genera Gp4, Gp5 and Gp6 (Table 2; Fig 3), which was consistent with the study of Liu et al. (2014) [83]. The irregular changes in acidobacterial abundance in the soil samples suggested there were some other factors, such as root exudates, that might affect the composition of bacterial communities in the soils.

### Complex microbial associations within the rhizospheric soil ecosystem

The co-occurrence patterns were examined by network analysis, and a total of 415 pairs of significant and robust correlations (positive and negative) were identified from 89 genera. Analyzing the co-occurrence patterns across the complex and diverse communities may help to understand the environmental niches and synergetic relationships in tree rhizospheric soils of the Taihu area [49]. There were two modules in the positive network, including the module of *Firmicutes* and *Bacteroidetes*, and the module of *Proteobacteria* and *Acidobacteria*. The coexistence of *Firmicutes* and *Bacteroidetes* and of *Proteobacteria* and *Acidobacteria* in the rhizospheric soils implied minimal competition for resources through cooperation or specialization. For example, Kampmann et al. (2012) reported that *Firmicutes* and *Bacteroidetes* communities were stable and hardly influenced in biogas reactors and that the members of *Firmicutes* favored lipid nutrients, whereas *Bacteroidetes* preferred starch nutrients [84]. A similar finding is that the phyla *Firmicutes* and *Bacteroidetes* are beneficial and dominant bacteria in the human gut. The relative abundance of *Bacteroidetes* is reduced in obese people in comparison with lean people, but this proportion increases along with weight loss on a low-calorie diet [85]. On the other hand, *Proteobacteria* and *Acidobacteria* are dominant phyla and are widely distributed across a range of ecosystems [17, 73–75, 86]. The members of *Acidobacteria* produce a wide range of enzymes with high activities under acidic conditions, whereas most of them grow along a narrow carbon resource spectrum, including disaccharides and oligosaccharides produced by the decomposition of cellulose, chitin and starch [87, 88]. Similarly, the *Proteobacteria* group also plays a key role in organic matter decomposition [88–90] by producing many kinds of glycosyl hydrolases, such as cellulases, chitinases, xylanases and amylases [91–94], and then generating a large number of oligosaccharides and aromatic alcohols, which can be used as carbon resource by other bacteria, such as *Acidobacteria*. Overall, *Acidobacteria* may play an important ecological role by collaboration with other microorganisms (the members of *Proteobacteria*) in the process of degrading polysaccharides of plant and fungal origin [88, 95].

### Effect of environmental variables on the soil microbial community

Previous studies have reported pH is strongly correlated with community composition [96, 97]. We also found pH was an integrated biogeochemical factor and was related to microbial structure and diversity. The diversity of bacterial communities increased and stabilized along with pH (3.84 to 7.79) in rhizospheric soils (S3 Fig). That is, lower pH was associated with less diverse communities [96]. This result was consistent with some previous studies that reported there was a trend of changes in bacterial diversity following a soil pH gradient (pHs of 3 to 9) [97, 98].

The rhizospheric soil microbial community structures were similar between evergreen trees and deciduous trees at the phylum level (Fig 2), which was consistent with some studies [27, 59, 96]. Dispersal limitation is known to be less important for microorganisms [70, 71], resulting in biogeographic patterns that primarily reflect selection by contemporary environmental conditions [99]. Overall, our results suggest that the soil bacterial community composition in the Taihu Lake area is determined more by local environmental conditions than by the biogeochemical characteristics of the rhizosphere. However, dispersal limitation may also be important in structuring the microbial taxa at a more precise level of taxonomy [100, 101]. Our results also showed there were some differences among the different rhizospheric samples at generic level (Fig 3), which may be indirectly driven by the plants. Some studies report that different plant species secrete different carbon compounds under the same conditions [61, 102], which leads to some changes in the soil microbial community structure [103]. In our study, we found that different tree species favored different nutrients and then induced some changes in the soil physic-chemical characteristics. For example, the soil pH level of the evergreen tree group was lower than that of the deciduous tree group; meanwhile, the content of NH_4_^+^ in the evergreen tree soils was lower than that for deciduous trees, while NO_3_^−^ was higher, which led to a lower pH in the evergreen tree rhizospheric soils (S3 Fig; Table 2). The relationship between pH and nitrogen resources suggested that the balance of nitrogen metabolism might impact the bacterial diversity of the rhizosphere. The adjustment of rhizospheric acidity is one of the most important methods for improving the availability of phosphorus and other metal ions in rhizosphere. For example, some coniferous species (such as *Picea abies*) can promote soil acidification and decreased pH [104]. Evergreen trees have a longer photosynthetic season than deciduous trees, therefore, rhizospheric acidification by evergreen trees could increase the absorbance of phosphorus and other metal ions.

Overall, our results indicated that the soil pH and tree species were the main influencing factors of the bacterial community composition and diversity in the Taihu Lake area, and the effect of pH was the key factor. Meanwhile, different plant species directly or indirectly affected the microbial communities by their root exudates and nutrient absorbance. Additionally, in the stable environments, *Proteobacteria* and *Acidobacteria* as well as *Firmicutes* and *Bacteroidetes* clustered into two modules, indicating there was some co-operation and specialization among the members of each module. In general, our results suggested that the compositional structure of bacterial communities in the Taihu Lake area was fundamentally determined by local environmental conditions associated with variation in soil acidity and plant species.

## Supporting information

S1 Fig Rarefaction curves based on the OTUs at the cutoff of 97% 16S rRNA sequence similarity. (TIF)

S2 Fig Abundances of different phyla associated with two types of trees. The taxa represented occurred at >1% abundance in association with at least one tree type.
(TIF)

S3 Fig Relationships between rhizospheric soil sample biogeochemical attributes and rhizospheric soil sample microbial community diversity (a); Relationships between rhizospheric soil sample pH and other biogeochemical attributes (b). (TIF)

S1 Table Relative abundances (% of total good-quality sequences) of all phyla in each sample across different tree species. The eleven phyla shaded blue were all the dominant phyla (>1% of good quality sequences in at least one sample), and their total abundances in each soil sample are shown in the second line from the bottom.
(DOCX)

S2 Table Relative abundances (% of total good-quality sequences) of all classified classes in each rhizospheric sample across different tree species. Forty dominant classes (shaded blue) were shared by all samples, and their total abundances in each sample are shown in the last line.
(DOCX)

S3 Table Relative abundances (% of total good-quality sequences) of all classified classes in each rhizospheric sample. The dominant classes (>5% of total good-quality sequences) of evergreen tree soil samples are shaded green, and those of deciduous tree soil samples are shaded yellow. Four classes of *Acidobacteria* were only shared by deciduous tree soil samples and are shaded pink. The total abundances of the dominant classes in each sample are shown in the last line.
(DOCX)

S4 Table Relative abundance of total shared genera (% of all classified genera/sequences) in each sample. (DOCX)

S5 Table Relative abundance of genera (% of all classified genera/sequences) in only one soil sample (DOCX)

S6 Table Relative abundances (% of total good-quality sequences) of twelve dominant genera (>1% of the classified sequences) in at least 5 soil samples. The percentages (% of total good-quality sequences) of 12 dominant genera are shown in the last line.
(DOCX)

S7 Table Relative abundances (>1% of total classified sequences) of dominant genera in each evergreen tree soil sample. (DOCX)

S8 Table Relative abundances (>1% of total classified sequences) of dominant genera in each deciduous tree soil sample. (DOCX)
