# Supplementary figures and images for: The rhizospheric microbial community structure and diversity of deciduous and evergreen forests in Taihu Lake area, China
Source: PLoS One. 2017 Apr 5;12(4):e0174411. doi: 10.1371/journal.pone.0174411 (PMC5381875; doi:10.1371/journal.pone.0174411)

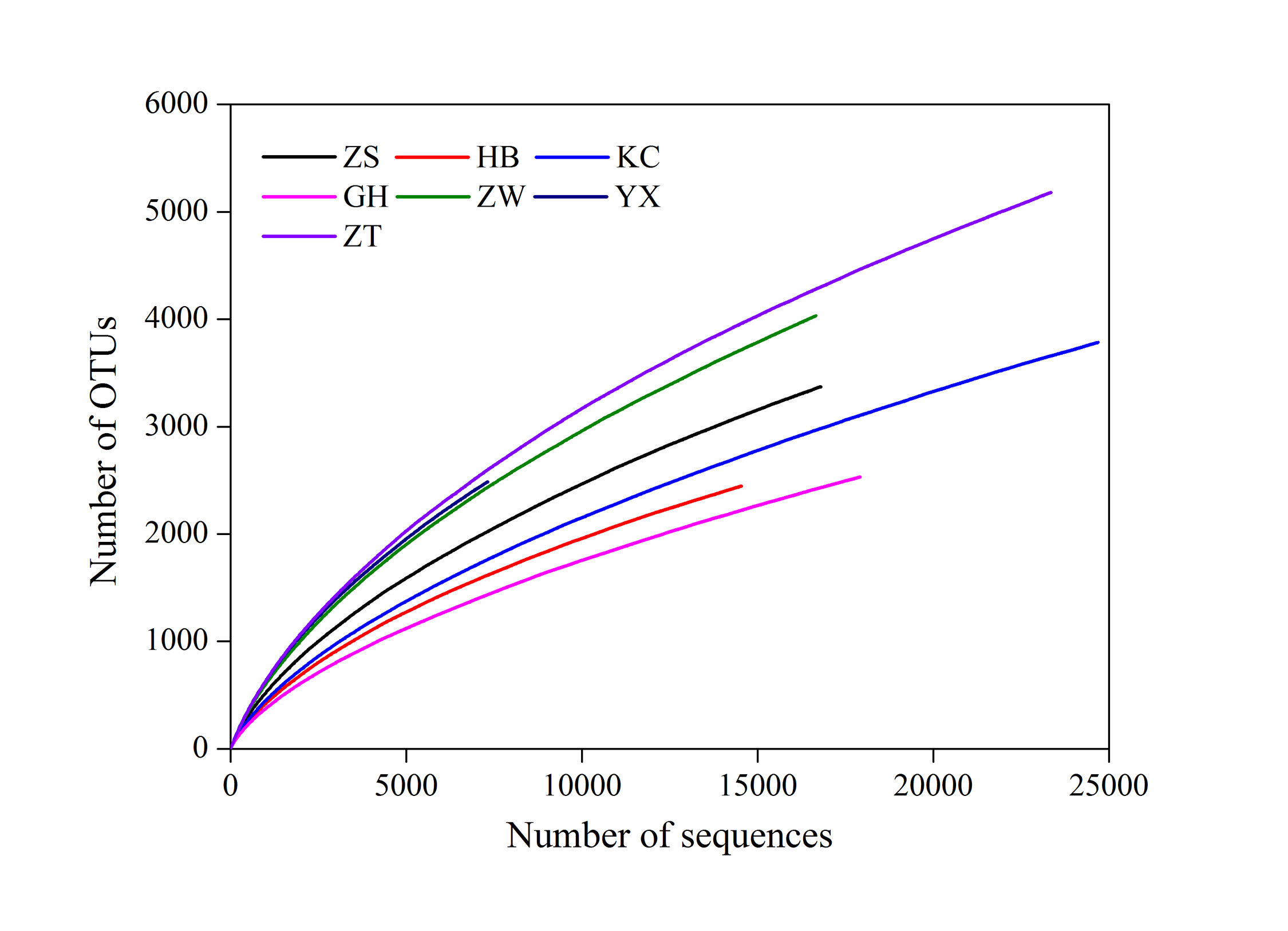

Supplement: S1 Fig — (TIF) [file pone.0174411.s001.tif]

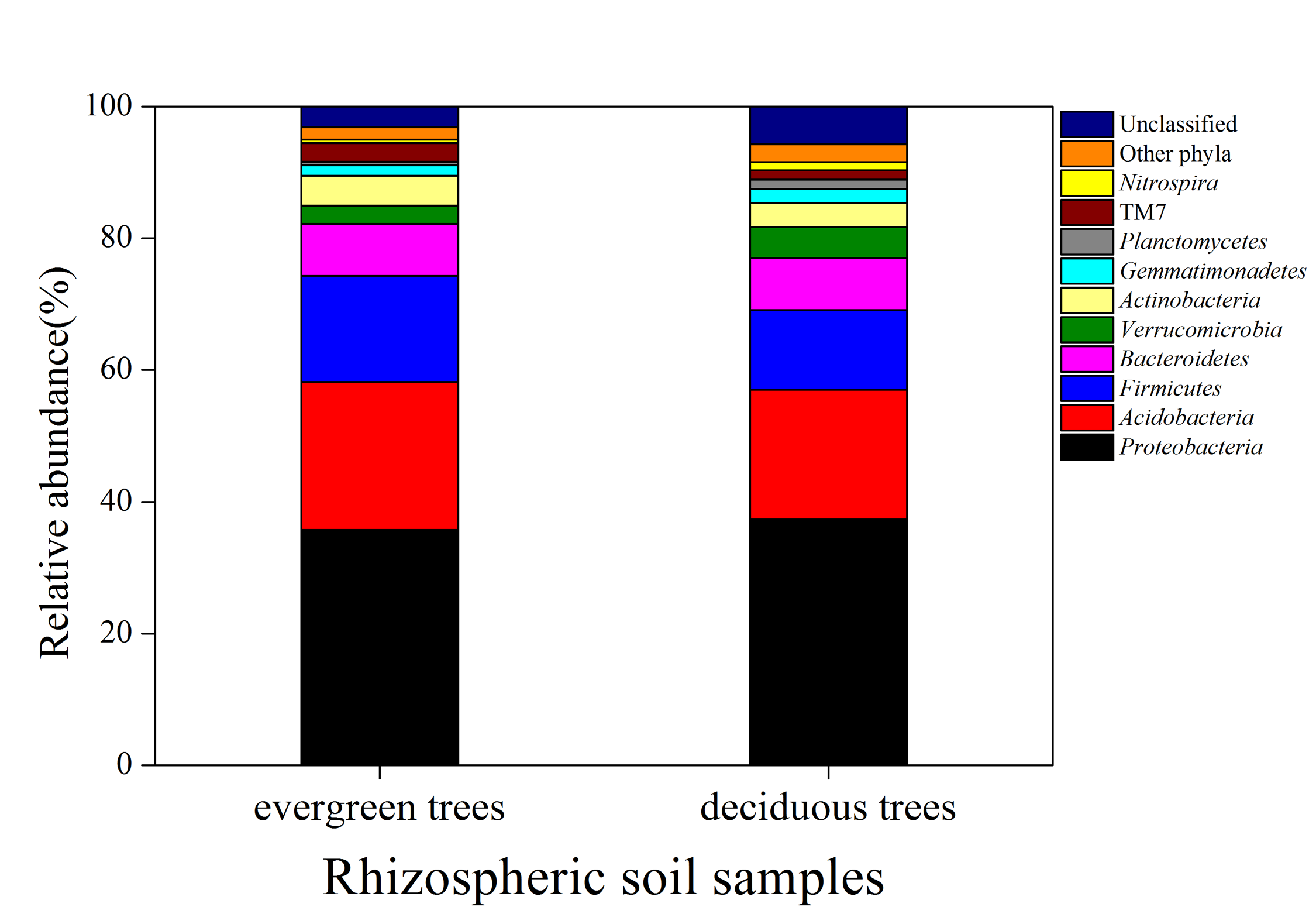

Supplement: S2 Fig — The taxa represented occurred at >1% abundance in association with at least one tree type. (TIF) [file pone.0174411.s002.tif]

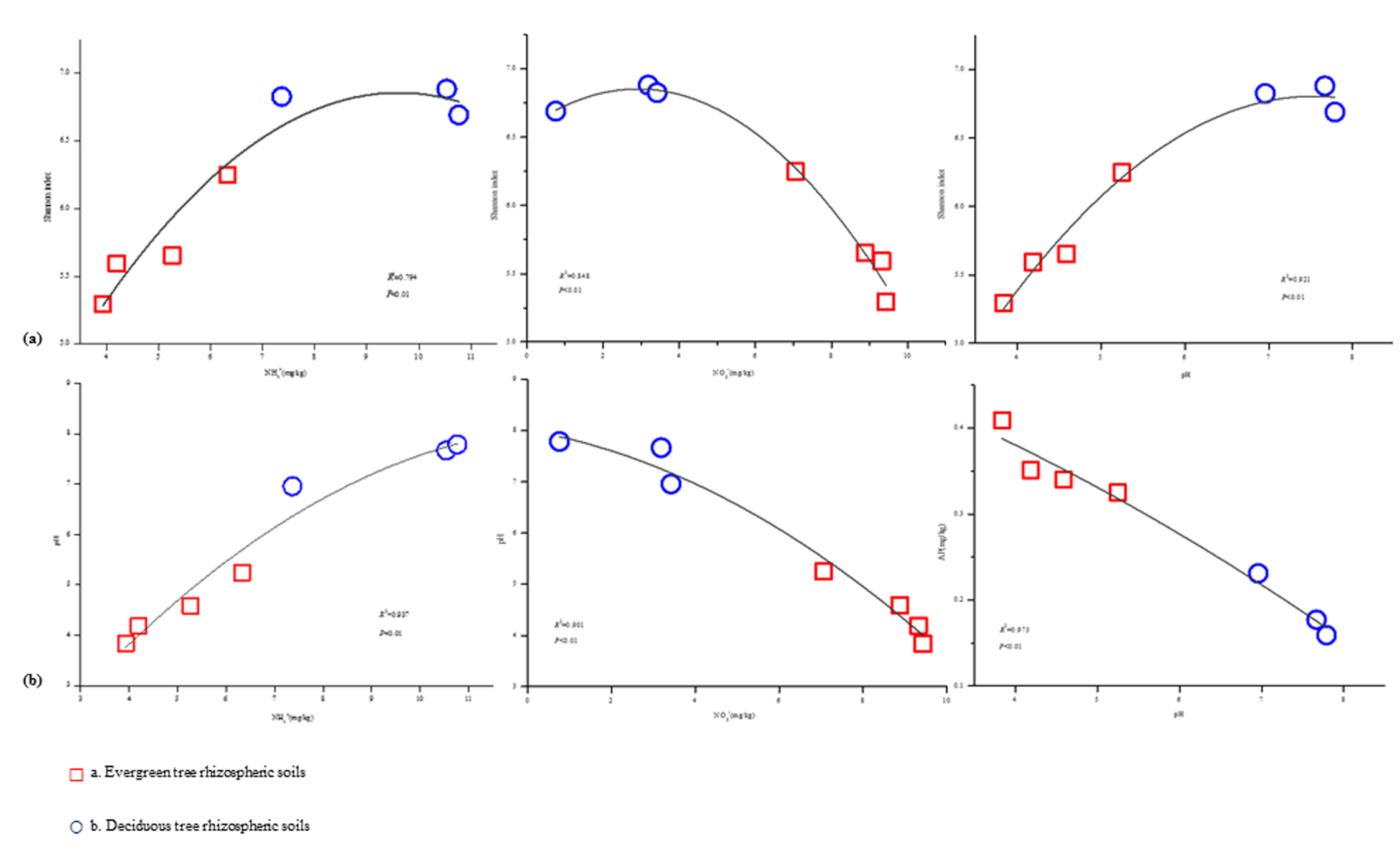

Supplement: S3 Fig — (TIF) [file pone.0174411.s003.tif]
