# Supplementary material for: The rhizospheric microbial community structure and diversity of deciduous and evergreen forests in Taihu Lake area, China
Source: PLoS One. 2017 Apr 5;12(4):e0174411. doi: 10.1371/journal.pone.0174411 (PMC5381875; doi:10.1371/journal.pone.0174411)
Supplement: S1 Table — The eleven phyla shaded blue were all the dominant phyla (>1% of good quality sequences in at least one sample), and their total abundances in each soil sample are shown in the second line from the bottom. (DOCX) [file pone.0174411.s004.docx]

**S1 Table.** **Relative abundances (% of total good-quality sequences) of all phyla in each sample across different tree species.** The eleven phyla shaded blue were all the dominant phyla (>1% of good quality sequences in at least one sample), and their total abundances in each soil sample are shown in the second line from the bottom.

| **Phylum** | **ZS** | **GH** | **HB** | **KC** | **ZW** | **YX** | **ZT** |
| --- | --- | --- | --- | --- | --- | --- | --- |
| *Proteobacteria* | 34.08 | 32.23 | 37.86 | 38.25 | 35.44 | 38.24 | 38.45 |
| *Acidobacteria* | 17.86 | 28.78 | 23.96 | 20.05 | 17.55 | 15.40 | 22.45 |
| *Firmicutes* | 14.85 | 14.70 | 14.29 | 19.03 | 11.59 | 17.15 | 10.92 |
| *Bacteroidetes* | 6.43 | 8.15 | 7.07 | 9.20 | 9.57 | 10.98 | 5.75 |
| *Actinobacteria* | 5.04 | 5.63 | 7.47 | 1.65 | 4.08 | 2.26 | 3.83 |
| *Verrucomicrobia* | 7.61 | 0.39 | 1.37 | 2.04 | 7.02 | 4.12 | 3.20 |
| *Gemmatimonadetes* | 4.06 | 1.06 | 0.72 | 0.85 | 1.87 | 1.31 | 2.57 |
| TM7 | 1.91 | 3.42 | 2.22 | 3.32 | 2.11 | 1.66 | 0.82 |
| *Nitrospira* | 1.10 | 0.06 | 0.06 | 0.74 | 1.18 | 1.08 | 1.36 |
| *Chloroflexi* | 0.71 | 1.66 | 0.25 | 0.75 | 0.33 | 0.41 | 1.22 |
| *Planctomycetes* | 0.71 | 0.51 | 0.84 | 0.31 | 2.20 | 0.67 | 1.15 |
| *Chlamydiae* | 0.56 | 0.04 | 0.34 | 0.28 | 0.25 | 0.55 | 0.46 |
| *Fusobacteria* | 0.21 | 0.11 | 0.50 | 0.13 | 0.34 | 0.79 | 0.37 |
| *Euryarchaeota* | 0.12 | 0.18 | 0.08 | 0.19 | 0.14 | 0.14 | 0.05 |
| OD1 | 0.11 | 0.01 | 0.08 | 0.06 | 0.11 | 0.05 | 0.08 |
| *Elusimicrobia* | 0.07 | 0.01 | 0.08 | 0.11 | 0.15 | 0.07 | 0.15 |
| *Armatimonadetes* | 0.07 | 0.02 | 0.10 | 0.07 | 0.07 | 0.05 | 0.07 |
| WS3 | 0.04 | 0.00 | 0.03 | 0.00 | 0.37 | 0.22 | 0.54 |
| *Chlorobi* | 0.04 | 0.02 | 0.01 | 0.02 | 0.02 | 0.12 | 0.08 |
| *Spirochaetes* | 0.03 | 0.04 | 0.06 | 0.06 | 0.04 | 0.12 | 0.08 |
| *Fibrobacteres* | 0.02 | 0.01 | 0.00 | 0.00 | 0.01 | 0.01 | 0.01 |
| *Cyanobacteria/Chloroplast* | 0.02 | 0.02 | 0.02 | 0.01 | 0.05 | 0.04 | 0.02 |
| *Tenericutes* | 0.01 | 0.01 | 0.01 | 0.02 | 0.01 | 0.11 | 0.03 |
| BRC1 | 0.01 | 0.00 | 0.00 | 0.00 | 0.02 | 0.00 | 0.03 |
| SR1 | 0.01 | 0.00 | 0.00 | 0.00 | 0.00 | 0.00 | 0.00 |
| *Deinococcus-Thermus* | 0.01 | 0.01 | 0.01 | 0.00 | 0.00 | 0.01 | 0.00 |
| *Synergistetes* | 0.00 | 0.08 | 0.00 | 0.01 | 0.00 | 0.01 | 0.00 |
| *Crenarchaeota* | 0.00 | 0.01 | 0.01 | 0.01 | 0.01 | 0.01 | 0.02 |
| OP11 | 0.00 | 0.00 | 0.00 | 0.00 | 0.00 | 0.00 | 0.01 |
| *Aquificae* | 0.00 | 0.00 | 0.00 | 0.00 | 0.01 | 0.00 | 0.01 |
| *Deferribacteres* | 0.00 | 0.00 | 0.00 | 0.00 | 0.01 | 0.00 | 0.00 |
| **The percentage of dominant phyla** | **94.36** | **96.58** | **96.09** | **96.19** | **92.93** | **93.28** | **91.72** |
| **The percentage of total identified phyla** | **95.68** | **97.15** | **97.40** | **97.15** | **94.54** | **95.61** | **93.74** |
