# Supplementary material for: The rhizospheric microbial community structure and diversity of deciduous and evergreen forests in Taihu Lake area, China
Source: PLoS One. 2017 Apr 5;12(4):e0174411. doi: 10.1371/journal.pone.0174411 (PMC5381875; doi:10.1371/journal.pone.0174411)
Supplement: S2 Table — Forty dominant classes (shaded blue) were shared by all samples, and their total abundances in each sample are shown in the last line. (DOCX) [file pone.0174411.s005.docx]

**S2 Table.** **Relative abundances (% of total good-quality sequences) of all classified classes in each rhizospheric sample across different tree species.** Forty dominant classes (shaded blue) were shared by all samples, and their total abundances in each sample are shown in the last line.

| **Class** | **GH** | **HB** | **KC** | **ZS** | **YX** | **ZT** | **ZW** |
| --- | --- | --- | --- | --- | --- | --- | --- |
| *Gammaproteobacteria* | 25.69 | 27.55 | 27.98 | 19.58 | 16.01 | 17.54 | 15.91 |
| *Clostridia* | 8.74 | 9.87 | 12.19 | 9.02 | 12.04 | 6.50 | 6.93 |
| *Alphaproteobacteria* | 5.16 | 7.13 | 6.66 | 8.15 | 12.02 | 9.16 | 8.01 |
| *Acidobacteria*_Gp2 | 17.60 | 10.21 | 5.42 | 3.34 | 0.29 | 4.71 | 0.07 |
| *Acidobacteria*_Gp1 | 8.63 | 8.84 | 8.44 | 8.06 | 0.70 | 2.13 | 0.10 |
| *Bacteroidia* | 6.60 | 5.38 | 7.06 | 4.82 | 4.12 | 3.15 | 3.75 |
| *Actinobacteria* | 5.63 | 7.47 | 1.65 | 5.04 | 2.26 | 3.83 | 4.08 |
| *Betaproteobacteria* | 0.83 | 1.33 | 1.67 | 3.23 | 5.60 | 5.01 | 6.37 |
| *Bacilli* | 2.41 | 1.60 | 2.49 | 2.61 | 2.12 | 2.32 | 2.02 |
| *Acidobacteria*_Gp3 | 0.98 | 1.95 | 4.09 | 2.74 | 1.56 | 1.03 | 1.05 |
| *Acidobacteria*_Gp6 | 0.07 | 0.29 | 0.71 | 0.92 | 6.61 | 6.49 | 8.73 |
| *Deltaproteobacteria* | 0.49 | 1.00 | 1.65 | 2.03 | 3.90 | 6.26 | 4.40 |
| *Sphingobacteria* | 0.30 | 0.51 | 0.56 | 0.46 | 3.60 | 1.15 | 3.18 |
| *Acidobacteria*_Gp4 | 0.05 | 0.15 | 0.13 | 0.82 | 2.35 | 2.52 | 3.51 |
| *Gemmatimonadetes* | 1.06 | 0.72 | 0.85 | 4.06 | 1.31 | 2.57 | 1.87 |
| *Subdivision3* | 0.33 | 1.04 | 1.48 | 2.01 | 2.13 | 1.99 | 3.28 |
| *Spartobacteria* | 0.03 | 0.15 | 0.47 | 5.47 | 1.40 | 1.02 | 3.20 |
| *Negativicutes* | 1.06 | 1.49 | 1.41 | 1.18 | 0.92 | 0.87 | 0.96 |
| *Bacteroidetes* | 0.54 | 0.52 | 0.72 | 0.54 | 1.15 | 0.69 | 1.44 |
| *Nitrospira* | 0.06 | 0.06 | 0.74 | 1.10 | 1.08 | 1.36 | 1.18 |
| *Acidobacteria*_Gp13 | 1.41 | 2.19 | 0.73 | 0.61 | 0.04 | 0.45 | 0.02 |
| *Planctomycetacia* | 0.43 | 0.70 | 0.20 | 0.59 | 0.52 | 0.82 | 1.77 |
| *Flavobacteria* | 0.49 | 0.41 | 0.53 | 0.40 | 1.08 | 0.39 | 0.60 |
| *Ktedonobacteria* | 1.59 | 0.14 | 0.56 | 0.52 | 0.07 | 0.38 | 0.04 |
| *Erysipelotrichia* | 0.36 | 0.32 | 0.60 | 0.43 | 0.62 | 0.30 | 0.44 |
| *Chlamydiae* | 0.04 | 0.34 | 0.28 | 0.56 | 0.55 | 0.46 | 0.25 |
| *Fusobacteria* | 0.11 | 0.50 | 0.13 | 0.21 | 0.79 | 0.37 | 0.34 |
| *Acidobacteria*_Gp7 | 0.01 | 0.03 | 0.12 | 0.23 | 0.22 | 0.84 | 0.68 |
| *Acidobacteria*_Gp16 | 0.02 | 0.04 | 0.03 | 0.41 | 0.22 | 0.26 | 0.58 |
| *Phycisphaerae* | 0.08 | 0.14 | 0.11 | 0.12 | 0.15 | 0.33 | 0.43 |
| *Acidobacteria*_Gp10 | 0.01 | 0.15 | 0.02 | 0.01 | 0.27 | 0.67 | 0.20 |
| *Opitutae* | 0.02 | 0.16 | 0.07 | 0.12 | 0.21 | 0.09 | 0.22 |
| *Anaerolineae* | 0.02 | 0.06 | 0.03 | 0.07 | 0.16 | 0.39 | 0.14 |
| *Verrucomicrobiae* | 0.01 | 0.01 | 0.01 | 0.01 | 0.34 | 0.09 | 0.31 |
| *Methanobacteria* | 0.09 | 0.07 | 0.19 | 0.12 | 0.14 | 0.04 | 0.14 |
| *Elusimicrobia* | 0.01 | 0.08 | 0.11 | 0.07 | 0.07 | 0.15 | 0.15 |
| *Spirochaetes* | 0.04 | 0.06 | 0.06 | 0.03 | 0.12 | 0.08 | 0.04 |
| *Epsilonproteobacteria* | 0.03 | 0.06 | 0.03 | 0.07 | 0.16 | 0.04 | 0.04 |
| *Ignavibacteria* | 0.01 | 0.01 | 0.02 | 0.03 | 0.12 | 0.08 | 0.02 |
| *Mollicutes* | 0.01 | 0.01 | 0.02 | 0.01 | 0.11 | 0.03 | 0.01 |
| *Chloroplast* | 0.02 | 0.02 | 0.00 | 0.02 | 0.03 | 0.02 | 0.05 |
| *Acidobacteria*_Gp5 | 0.00 | 0.02 | 0.18 | 0.20 | 1.34 | 1.17 | 1.02 |
| *Acidobacteria*_Gp25 | 0.01 | 0.00 | 0.00 | 0.25 | 0.53 | 0.56 | 0.57 |
| *Acidobacteria*_Gp11 | 0.00 | 0.00 | 0.00 | 0.01 | 0.62 | 0.47 | 0.40 |
| *Acidobacteria*_Gp22 | 0.00 | 0.00 | 0.00 | 0.00 | 0.33 | 0.43 | 0.17 |
| *Acidobacteria*_Gp17 | 0.01 | 0.01 | 0.00 | 0.05 | 0.21 | 0.28 | 0.28 |
| *Acidobacteria*_Gp15 | 0.00 | 0.00 | 0.15 | 0.17 | 0.04 | 0.24 | 0.08 |
| *Dehalococcoidetes* | 0.01 | 0.00 | 0.10 | 0.08 | 0.04 | 0.22 | 0.04 |
| *Thermomicrobia* | 0.02 | 0.05 | 0.01 | 0.00 | 0.07 | 0.16 | 0.06 |
| *Caldilineae* | 0.01 | 0.00 | 0.02 | 0.01 | 0.05 | 0.06 | 0.04 |
| *Acidobacteria*_Gp9 | 0.00 | 0.00 | 0.00 | 0.00 | 0.03 | 0.12 | 0.04 |
| *Synergistia* | 0.08 | 0.00 | 0.01 | 0.00 | 0.01 | 0.00 | 0.00 |
| *Acidobacteria*_Gp18 | 0.00 | 0.00 | 0.00 | 0.00 | 0.04 | 0.03 | 0.02 |
| *Thermoplasmata* | 0.08 | 0.00 | 0.00 | 0.00 | 0.00 | 0.00 | 0.00 |
| *Acidobacteria*_Gp14 | 0.00 | 0.08 | 0.00 | 0.00 | 0.00 | 0.00 | 0.00 |
| *Fibrobacteria* | 0.01 | 0.00 | 0.00 | 0.02 | 0.01 | 0.01 | 0.01 |
| *Thermoprotei* | 0.01 | 0.01 | 0.01 | 0.00 | 0.01 | 0.02 | 0.01 |
| *Subdivision5* | 0.00 | 0.01 | 0.00 | 0.00 | 0.04 | 0.00 | 0.01 |
| *Acidobacteria*_Gp20 | 0.00 | 0.00 | 0.00 | 0.00 | 0.01 | 0.03 | 0.01 |
| *Acidobacteria*_Gp12 | 0.00 | 0.00 | 0.01 | 0.02 | 0.00 | 0.02 | 0.00 |
| *Deinococci* | 0.01 | 0.01 | 0.00 | 0.01 | 0.01 | 0.00 | 0.00 |
| *Holophagae* | 0.00 | 0.01 | 0.02 | 0.01 | 0.00 | 0.00 | 0.00 |
| *Cyanobacteria* | 0.00 | 0.00 | 0.00 | 0.00 | 0.01 | 0.00 | 0.01 |
| *Methanomicrobia* | 0.01 | 0.01 | 0.00 | 0.00 | 0.00 | 0.00 | 0.00 |
| *Aquificae* | 0.00 | 0.00 | 0.00 | 0.00 | 0.00 | 0.01 | 0.01 |
| *Chlorobia* | 0.01 | 0.00 | 0.00 | 0.01 | 0.00 | 0.00 | 0.00 |
| *Chloroflexi* | 0.00 | 0.00 | 0.00 | 0.01 | 0.00 | 0.00 | 0.01 |
| *Deferribacteres* | 0.00 | 0.00 | 0.00 | 0.00 | 0.00 | 0.00 | 0.01 |
| **The percentages of the classes shared by all soil samples** | **91.01** | **92.71** | **90.21** | **89.81** | **87.11** | **86.56** | **86.48** |
