# Supplementary material for: The rhizospheric microbial community structure and diversity of deciduous and evergreen forests in Taihu Lake area, China
Source: PLoS One. 2017 Apr 5;12(4):e0174411. doi: 10.1371/journal.pone.0174411 (PMC5381875; doi:10.1371/journal.pone.0174411)
Supplement: S3 Table — The dominant classes (>5% of total good-quality sequences) of evergreen tree soil samples are shaded green, and those of deciduous tree soil samples are shaded yellow. Four classes of Acidobacteria were only shared by deciduous tree soil samples and are shaded pink. The total abundances of the dominant classes in each sample are shown in the last line. (DOCX) [file pone.0174411.s006.docx]

**S3 Table.** **Relative abundances (% of total good-quality sequences) of all classified classes in each rhizospheric sample.** The dominant classes (>5% of total good-quality sequences) of evergreen tree soil samples are shaded green, and those of deciduous tree soil samples are shaded yellow. Four classes of *Acidobacteria* were only shared by deciduous tree soil samples and are shaded pink. The total abundances of the dominant classes in each sample are shown in the last line.

| **Class** | **GH** | **HB** | **KC** | **ZS** | **YX** | **ZT** | **ZW** |
| --- | --- | --- | --- | --- | --- | --- | --- |
| *Gammaproteobacteria* | 25.69 | 27.55 | 27.98 | 19.58 | 16.01 | 17.54 | 15.91 |
| *Clostridia* | 8.74 | 9.87 | 12.19 | 9.02 | 12.04 | 6.50 | 6.93 |
| *Alphaproteobacteria* | 5.16 | 7.13 | 6.66 | 8.15 | 12.02 | 9.16 | 8.01 |
| *Acidobacteria*_Gp2 | 17.60 | 10.21 | 5.42 | 3.34 | 0.29 | 4.71 | 0.07 |
| *Acidobacteria*_Gp1 | 8.63 | 8.84 | 8.44 | 8.06 | 0.70 | 2.13 | 0.10 |
| *Betaproteobacteria* | 0.83 | 1.33 | 1.67 | 3.23 | 5.60 | 5.01 | 6.37 |
| *Acidobacteria*_Gp6 | 0.07 | 0.29 | 0.71 | 0.92 | 6.61 | 6.49 | 8.73 |
| *Deltaproteobacteria* | 0.49 | 1.00 | 1.65 | 2.03 | 3.90 | 6.26 | 4.40 |
| *Acidobacteria*_Gp3 | 0.98 | 1.95 | 4.09 | 2.74 | 1.56 | 1.03 | 1.05 |
| *Acidobacteria*_Gp4 | 0.05 | 0.15 | 0.13 | 0.82 | 2.35 | 2.52 | 3.51 |
| *Acidobacteria*_Gp5 | 0.00 | 0.02 | 0.18 | 0.20 | 1.34 | 1.17 | 1.02 |
| *Acidobacteria*_Gp7 | 0.01 | 0.03 | 0.12 | 0.23 | 0.22 | 0.84 | 0.68 |
| *Acidobacteria*_Gp9 | 0.00 | 0.00 | 0.00 | 0.00 | 0.03 | 0.12 | 0.04 |
| *Acidobacteria*_Gp18 | 0.00 | 0.00 | 0.00 | 0.00 | 0.04 | 0.03 | 0.02 |
| *Acidobacteria*_Gp20 | 0.00 | 0.00 | 0.00 | 0.00 | 0.01 | 0.03 | 0.01 |
| *Acidobacteria*_Gp22 | 0.00 | 0.00 | 0.00 | 0.00 | 0.33 | 0.43 | 0.17 |
| *Acidobacteria*_Gp10 | 0.01 | 0.15 | 0.02 | 0.01 | 0.27 | 0.67 | 0.20 |
| *Acidobacteria*_Gp11 | 0.00 | 0.00 | 0.00 | 0.01 | 0.62 | 0.47 | 0.40 |
| *Acidobacteria*_Gp12 | 0.00 | 0.00 | 0.01 | 0.02 | 0.00 | 0.02 | 0.00 |
| *Acidobacteria*_Gp13 | 1.41 | 2.19 | 0.73 | 0.61 | 0.04 | 0.45 | 0.02 |
| *Acidobacteria*_Gp14 | 0.00 | 0.08 | 0.00 | 0.00 | 0.00 | 0.00 | 0.00 |
| *Acidobacteria*_Gp15 | 0.00 | 0.00 | 0.15 | 0.17 | 0.04 | 0.24 | 0.08 |
| *Acidobacteria*_Gp16 | 0.02 | 0.04 | 0.03 | 0.41 | 0.22 | 0.26 | 0.58 |
| *Acidobacteria*_Gp17 | 0.01 | 0.01 | 0.00 | 0.05 | 0.21 | 0.28 | 0.28 |
| *Acidobacteria*_Gp25 | 0.01 | 0.00 | 0.00 | 0.25 | 0.53 | 0.56 | 0.57 |
| *Bacteroidia* | 6.60 | 5.38 | 7.06 | 4.82 | 4.12 | 3.15 | 3.75 |
| *Actinobacteria* | 5.63 | 7.47 | 1.65 | 5.04 | 2.26 | 3.83 | 4.08 |
| *Bacilli* | 2.41 | 1.60 | 2.49 | 2.61 | 2.12 | 2.32 | 2.02 |
| *Sphingobacteria* | 0.30 | 0.51 | 0.56 | 0.46 | 3.60 | 1.15 | 3.18 |
| *Gemmatimonadetes* | 1.06 | 0.72 | 0.85 | 4.06 | 1.31 | 2.57 | 1.87 |
| *Subdivision3* | 0.33 | 1.04 | 1.48 | 2.01 | 2.13 | 1.99 | 3.28 |
| *Spartobacteria* | 0.03 | 0.15 | 0.47 | 5.47 | 1.40 | 1.02 | 3.20 |
| *Negativicutes* | 1.06 | 1.49 | 1.41 | 1.18 | 0.92 | 0.87 | 0.96 |
| *Bacteroidetes* | 0.54 | 0.52 | 0.72 | 0.54 | 1.15 | 0.69 | 1.44 |
| *Nitrospira* | 0.06 | 0.06 | 0.74 | 1.10 | 1.08 | 1.36 | 1.18 |
| *Planctomycetacia* | 0.43 | 0.70 | 0.20 | 0.59 | 0.52 | 0.82 | 1.77 |
| *Flavobacteria* | 0.49 | 0.41 | 0.53 | 0.40 | 1.08 | 0.39 | 0.60 |
| *Ktedonobacteria* | 1.59 | 0.14 | 0.56 | 0.52 | 0.07 | 0.38 | 0.04 |
| *Erysipelotrichia* | 0.36 | 0.32 | 0.60 | 0.43 | 0.62 | 0.30 | 0.44 |
| *Chlamydiae* | 0.04 | 0.34 | 0.28 | 0.56 | 0.55 | 0.46 | 0.25 |
| *Fusobacteria* | 0.11 | 0.50 | 0.13 | 0.21 | 0.79 | 0.37 | 0.34 |
| *Phycisphaerae* | 0.08 | 0.14 | 0.11 | 0.12 | 0.15 | 0.33 | 0.43 |
| *Opitutae* | 0.02 | 0.16 | 0.07 | 0.12 | 0.21 | 0.09 | 0.22 |
| *Anaerolineae* | 0.02 | 0.06 | 0.03 | 0.07 | 0.16 | 0.39 | 0.14 |
| *Verrucomicrobiae* | 0.01 | 0.01 | 0.01 | 0.01 | 0.34 | 0.09 | 0.31 |
| *Methanobacteria* | 0.09 | 0.07 | 0.19 | 0.12 | 0.14 | 0.04 | 0.14 |
| *Elusimicrobia* | 0.01 | 0.08 | 0.11 | 0.07 | 0.07 | 0.15 | 0.15 |
| *Spirochaetes* | 0.04 | 0.06 | 0.06 | 0.03 | 0.12 | 0.08 | 0.04 |
| *Epsilonproteobacteria* | 0.03 | 0.06 | 0.03 | 0.07 | 0.16 | 0.04 | 0.04 |
| *Ignavibacteria* | 0.01 | 0.01 | 0.02 | 0.03 | 0.12 | 0.08 | 0.02 |
| *Mollicutes* | 0.01 | 0.01 | 0.02 | 0.01 | 0.11 | 0.03 | 0.01 |
| *Chloroplast* | 0.02 | 0.02 | 0.00 | 0.02 | 0.03 | 0.02 | 0.05 |
| *Dehalococcoidetes* | 0.01 | 0.00 | 0.10 | 0.08 | 0.04 | 0.22 | 0.04 |
| *Thermomicrobia* | 0.02 | 0.05 | 0.01 | 0.00 | 0.07 | 0.16 | 0.06 |
| *Caldilineae* | 0.01 | 0.00 | 0.02 | 0.01 | 0.05 | 0.06 | 0.04 |
| *Synergistia* | 0.08 | 0.00 | 0.01 | 0.00 | 0.01 | 0.00 | 0.00 |
| *Thermoplasmata* | 0.08 | 0.00 | 0.00 | 0.00 | 0.00 | 0.00 | 0.00 |
| *Fibrobacteria* | 0.01 | 0.00 | 0.00 | 0.02 | 0.01 | 0.01 | 0.01 |
| *Thermoprotei* | 0.01 | 0.01 | 0.01 | 0.00 | 0.01 | 0.02 | 0.01 |
| *Subdivision5* | 0.00 | 0.01 | 0.00 | 0.00 | 0.04 | 0.00 | 0.01 |
| *Deinococci* | 0.01 | 0.01 | 0.00 | 0.01 | 0.01 | 0.00 | 0.00 |
| *Holophagae* | 0.00 | 0.01 | 0.02 | 0.01 | 0.00 | 0.00 | 0.00 |
| *Cyanobacteria* | 0.00 | 0.00 | 0.00 | 0.00 | 0.01 | 0.00 | 0.01 |
| *Methanomicrobia* | 0.01 | 0.01 | 0.00 | 0.00 | 0.00 | 0.00 | 0.00 |
| *Aquificae* | 0.00 | 0.00 | 0.00 | 0.00 | 0.00 | 0.01 | 0.01 |
| *Chlorobia* | 0.01 | 0.00 | 0.00 | 0.01 | 0.00 | 0.00 | 0.00 |
| *Chloroflexi* | 0.00 | 0.00 | 0.00 | 0.01 | 0.00 | 0.00 | 0.01 |
| *Deferribacteres* | 0.00 | 0.00 | 0.00 | 0.00 | 0.00 | 0.00 | 0.01 |
| **The percentages of the dominant classes in each soil sample** | **65.81** | **63.60** | **60.69** | **48.16** | **52.27** | **44.69** | **45.95** |
