# Supplementary material for: The rhizospheric microbial community structure and diversity of deciduous and evergreen forests in Taihu Lake area, China
Source: PLoS One. 2017 Apr 5;12(4):e0174411. doi: 10.1371/journal.pone.0174411 (PMC5381875; doi:10.1371/journal.pone.0174411)
Supplement: S4 Table — (DOCX) [file pone.0174411.s007.docx]

**S4 Table. Relative abundance of total shared genera (% of all classified genera/sequences) in each sample**

| **Genus** | **Samples** | | | | | | |
| --- | --- | --- | --- | --- | --- | --- | --- |
|  | **ZS** | **GH** | **HB** | **KC** | **ZW** | **YX** | **ZT** |
| *Acetanaerobacterium* | 0.01 | 0.01 | 0.02 | 0.03 | 0.01 | 0.13 | 0.04 |
| *Acetivibrio* | 0.02 | 0.04 | 0.03 | 0.04 | 0.06 | 0.14 | 0.06 |
| *Aciditerrimonas* | 0.48 | 1.04 | 2.54 | 0.29 | 0.57 | 0.24 | 0.66 |
| *Acinetobacter* | 1.78 | 2.51 | 1.89 | 4.27 | 1.65 | 1.30 | 1.37 |
| *Actinocorallia* | 0.07 | 0.32 | 0.16 | 0.01 | 0.01 | 0.03 | 0.03 |
| *Actinomyces* | 0.03 | 0.01 | 0.04 | 0.01 | 0.01 | 0.03 | 0.05 |
| *Afipia* | 0.62 | 0.02 | 0.05 | 0.01 | 0.06 | 0.29 | 0.05 |
| *Allobaculum* | 0.03 | 0.01 | 0.02 | 0.04 | 0.04 | 0.05 | 0.02 |
| *Anaerophaga* | 0.35 | 0.16 | 0.19 | 0.30 | 0.17 | 0.18 | 0.12 |
| *Anaerotruncus* | 0.04 | 0.03 | 0.03 | 0.05 | 0.01 | 0.06 | 0.03 |
| *Anaerovibrio* | 0.05 | 0.03 | 0.01 | 0.06 | 0.04 | 0.05 | 0.04 |
| *Anaerovorax* | 0.04 | 0.04 | 0.04 | 0.09 | 0.06 | 0.06 | 0.04 |
| *Aquicella* | 0.66 | 0.07 | 0.07 | 0.15 | 0.38 | 0.18 | 0.32 |
| *Arenimonas* | 0.07 | 0.01 | 0.01 | 0.02 | 0.07 | 0.05 | 0.03 |
| *Armatimonadetes_gp5* | 0.07 | 0.02 | 0.09 | 0.07 | 0.08 | 0.05 | 0.08 |
| *Azohydromonas* | 0.01 | 0.01 | 0.01 | 0.05 | 0.10 | 0.35 | 0.13 |
| *Azospira* | 0.01 | 0.01 | 0.02 | 0.01 | 0.02 | 0.05 | 0.02 |
| *Bacillus* | 1.18 | 0.66 | 0.02 | 0.10 | 0.11 | 0.16 | 0.39 |
| *Bacteroides* | 0.09 | 0.07 | 0.07 | 0.11 | 0.09 | 0.06 | 0.02 |
| *Barnesiella* | 2.17 | 3.57 | 2.94 | 3.11 | 1.55 | 1.75 | 1.42 |
| *Bauldia* | 0.24 | 0.02 | 0.18 | 0.50 | 0.16 | 0.34 | 0.38 |
| *Bdellovibrio* | 0.04 | 0.04 | 0.06 | 0.01 | 0.08 | 0.03 | 0.03 |
| *Bellilinea* | 0.01 | 0.01 | 0.02 | 0.01 | 0.05 | 0.02 | 0.17 |
| *Bilophila* | 0.04 | 0.02 | 0.04 | 0.03 | 0.02 | 0.03 | 0.04 |
| *Blastopirellula* | 0.02 | 0.02 | 0.13 | 0.01 | 0.12 | 0.10 | 0.18 |
| *Blautia* | 0.11 | 0.04 | 0.07 | 0.09 | 0.12 | 0.18 | 0.06 |
| *Bradyrhizobium* | 0.41 | 0.48 | 0.54 | 0.27 | 0.29 | 0.35 | 0.17 |
| *Bulleidia* | 0.32 | 0.19 | 0.19 | 0.37 | 0.28 | 0.27 | 0.17 |
| *Burkholderia* | 1.13 | 0.44 | 0.25 | 0.19 | 0.04 | 0.02 | 0.16 |
| *Butyrivibrio* | 0.50 | 0.39 | 0.49 | 0.62 | 0.35 | 0.39 | 0.38 |
| *Byssovorax* | 0.15 | 0.03 | 0.08 | 0.08 | 0.35 | 0.14 | 0.25 |
| *Campylobacter* | 0.01 | 0.01 | 0.03 | 0.01 | 0.01 | 0.08 | 0.03 |
| *Catonella* | 0.03 | 0.01 | 0.04 | 0.05 | 0.03 | 0.05 | 0.03 |
| *Chryseobacterium* | 0.12 | 0.20 | 0.10 | 0.10 | 0.12 | 0.14 | 0.14 |
| *Citrobacter* | 0.12 | 0.13 | 0.10 | 0.23 | 0.09 | 0.06 | 0.11 |
| *Clostridium* IV | 0.31 | 0.24 | 0.31 | 0.49 | 0.29 | 0.68 | 0.30 |
| *Clostridium sensu stricto* | 0.05 | 0.03 | 0.01 | 0.02 | 0.01 | 0.13 | 0.03 |
| *Clostridium* XlVa | 1.02 | 0.80 | 0.80 | 1.30 | 0.79 | 1.43 | 0.63 |
| *Clostridium* XlVb | 0.06 | 0.03 | 0.07 | 0.03 | 0.06 | 0.05 | 0.03 |
| *Conexibacter* | 0.12 | 0.62 | 1.18 | 0.03 | 0.06 | 0.02 | 0.31 |
| *Coprococcus* | 0.01 | 0.05 | 0.13 | 0.07 | 0.02 | 0.11 | 0.04 |
| *Corynebacterium* | 0.02 | 0.01 | 0.05 | 0.03 | 0.01 | 0.03 | 0.02 |
| *Coxiella* | 0.10 | 0.01 | 0.08 | 0.03 | 0.07 | 0.16 | 0.08 |
| *Delftia* | 0.15 | 0.07 | 0.12 | 0.09 | 0.07 | 0.16 | 0.17 |
| *Denitratisoma* | 0.02 | 0.01 | 0.01 | 0.01 | 0.49 | 0.23 | 0.44 |
| *Desertibacter* | 0.04 | 0.04 | 0.02 | 0.01 | 0.07 | 0.03 | 0.02 |
| *Desulfomonile* | 0.16 | 0.17 | 0.08 | 0.30 | 0.02 | 0.18 | 0.31 |
| *Desulfovibrio* | 0.03 | 0.04 | 0.02 | 0.05 | 0.01 | 0.02 | 0.04 |
| *Dethiosulfatibacter* | 0.01 | 0.08 | 0.49 | 0.06 | 0.04 | 0.06 | 0.05 |
| *Devosia* | 0.03 | 0.01 | 0.09 | 0.00 | 0.04 | 0.08 | 0.08 |
| *Dongia* | 0.56 | 0.06 | 0.25 | 0.26 | 2.17 | 2.97 | 1.34 |
| *Dorea* | 0.02 | 0.02 | 0.01 | 0.02 | 0.03 | 0.14 | 0.03 |
| *Elusimicrobium* | 0.08 | 0.01 | 0.08 | 0.12 | 0.18 | 0.08 | 0.19 |
| *Enterococcus* | 0.10 | 0.16 | 0.07 | 0.21 | 0.12 | 0.08 | 0.09 |
| *Enterorhabdus* | 0.02 | 0.07 | 0.04 | 0.03 | 0.04 | 0.05 | 0.02 |
| *Escherichia/Shigella* | 0.25 | 0.15 | 0.55 | 0.21 | 0.22 | 0.29 | 0.48 |
| *Eubacterium* | 0.33 | 0.25 | 0.30 | 0.36 | 0.23 | 0.43 | 0.27 |
| *Faecalibacterium* | 1.16 | 0.98 | 0.99 | 1.54 | 0.76 | 1.30 | 0.79 |
| *Ferruginibacter* | 0.01 | 0.01 | 0.04 | 0.01 | 0.06 | 0.18 | 0.04 |
| *Flavihumibacter* | 0.04 | 0.01 | 0.02 | 0.00 | 0.12 | 0.11 | 0.06 |
| *Flavitalea* | 0.03 | 0.01 | 0.03 | 0.04 | 0.30 | 0.14 | 0.10 |
| *Flavonifractor* | 0.10 | 0.07 | 0.14 | 0.13 | 0.11 | 0.18 | 0.13 |
| *Fusobacterium* | 0.16 | 0.09 | 0.44 | 0.12 | 0.27 | 0.64 | 0.27 |
| *Galbibacter* | 0.30 | 0.32 | 0.31 | 0.45 | 0.22 | 0.10 | 0.20 |
| *Gemmata* | 0.28 | 0.19 | 0.31 | 0.05 | 1.08 | 0.19 | 0.27 |
| *Gemmatimonas* | 4.62 | 1.16 | 0.78 | 0.96 | 2.25 | 1.54 | 3.11 |
| *Gemmiger* | 0.16 | 0.10 | 0.12 | 0.17 | 0.14 | 0.32 | 0.09 |
| Gp1 | 8.84 | 8.77 | 8.96 | 8.79 | 0.10 | 0.82 | 2.34 |
| Gp10 | 0.01 | 0.01 | 0.16 | 0.02 | 0.24 | 0.32 | 0.81 |
| Gp13 | 0.69 | 1.53 | 2.38 | 0.82 | 0.02 | 0.05 | 0.54 |
| Gp16 | 0.47 | 0.02 | 0.04 | 0.03 | 0.70 | 0.26 | 0.31 |
| Gp2 | 3.81 | 19.17 | 11.12 | 6.07 | 0.09 | 0.34 | 5.70 |
| Gp3 | 3.12 | 1.07 | 2.12 | 4.58 | 1.26 | 1.82 | 1.25 |
| Gp4 | 0.94 | 0.05 | 0.16 | 0.15 | 4.22 | 2.77 | 3.06 |
| Gp6 | 1.05 | 0.07 | 0.31 | 0.80 | 10.48 | 7.77 | 7.85 |
| Gp7 | 0.26 | 0.01 | 0.04 | 0.13 | 0.82 | 0.26 | 1.02 |
| *Granulicatella* | 0.01 | 0.01 | 0.04 | 0.01 | 0.01 | 0.06 | 0.02 |
| *Haemophilus* | 0.05 | 0.01 | 0.16 | 0.04 | 0.04 | 0.08 | 0.11 |
| *Hallella* | 0.08 | 0.07 | 0.06 | 0.13 | 0.06 | 0.05 | 0.03 |
| *Helicobacter* | 0.02 | 0.02 | 0.03 | 0.02 | 0.03 | 0.11 | 0.02 |
| *Holdemania* | 0.13 | 0.17 | 0.10 | 0.18 | 0.12 | 0.24 | 0.13 |
| *Hydrogenophaga* | 0.04 | 0.05 | 0.10 | 0.02 | 0.01 | 0.10 | 0.06 |
| *Hyphomicrobium* | 0.03 | 0.01 | 0.07 | 0.01 | 0.06 | 0.11 | 0.11 |
| *Iamia* | 0.23 | 0.06 | 0.17 | 0.08 | 0.33 | 0.27 | 0.24 |
| *Ignavibacterium* | 0.03 | 0.01 | 0.01 | 0.02 | 0.03 | 0.14 | 0.09 |
| *Ilumatobacter* | 0.02 | 0.02 | 0.03 | 0.01 | 0.22 | 0.13 | 0.13 |
| *Isobaculum* | 0.01 | 0.01 | 0.01 | 0.01 | 0.02 | 0.02 | 0.01 |
| *Klebsiella* | 0.27 | 0.20 | 0.19 | 0.41 | 0.10 | 0.11 | 0.17 |
| *Kluyvera* | 0.24 | 0.32 | 0.47 | 0.14 | 0.26 | 0.14 | 0.13 |
| *Kofleria* | 0.47 | 0.01 | 0.13 | 0.39 | 0.64 | 0.69 | 0.90 |
| *Ktedonobacter* | 0.29 | 1.41 | 0.11 | 0.20 | 0.04 | 0.03 | 0.35 |
| *Kurthia* | 0.01 | 0.04 | 0.03 | 0.03 | 0.02 | 0.03 | 0.02 |
| *Lachnospiracea_incertae_sedis* | 1.47 | 1.36 | 1.26 | 1.95 | 1.14 | 1.48 | 0.89 |
| *Lactobacillus* | 0.29 | 0.33 | 0.31 | 0.51 | 0.31 | 0.69 | 0.41 |
| *Leptotrichia* | 0.09 | 0.04 | 0.10 | 0.03 | 0.14 | 0.27 | 0.18 |
| *Longilinea* | 0.07 | 0.01 | 0.04 | 0.02 | 0.05 | 0.13 | 0.19 |
| *Methanobrevibacter* | 0.14 | 0.10 | 0.07 | 0.21 | 0.17 | 0.14 | 0.05 |
| *Methylococcus* | 0.07 | 0.04 | 0.03 | 0.03 | 0.02 | 0.05 | 0.10 |
| *Methyloversatilis* | 0.02 | 0.01 | 0.01 | 0.01 | 0.02 | 0.02 | 0.06 |
| *Mycobacterium* | 0.04 | 0.06 | 0.08 | 0.02 | 0.01 | 0.05 | 0.02 |
| *Neochlamydia* | 0.32 | 0.03 | 0.31 | 0.21 | 0.19 | 0.39 | 0.23 |
| *Nitrospira* | 1.26 | 0.07 | 0.06 | 0.83 | 1.41 | 1.24 | 1.64 |
| OD1*_genera_incertae_sedis* | 0.12 | 0.01 | 0.09 | 0.06 | 0.14 | 0.06 | 0.09 |
| *Ohtaekwangia* | 0.05 | 0.01 | 0.04 | 0.06 | 1.26 | 0.96 | 0.46 |
| *Opitutus* | 0.12 | 0.01 | 0.17 | 0.05 | 0.24 | 0.16 | 0.10 |
| *Oribacterium* | 0.69 | 0.75 | 0.67 | 1.22 | 0.58 | 0.84 | 0.43 |
| *Oscillibacter* | 0.64 | 0.60 | 0.68 | 0.86 | 0.51 | 1.30 | 0.62 |
| *Paenibacillus* | 0.03 | 0.01 | 0.01 | 0.01 | 0.03 | 0.03 | 0.11 |
| *Paludibacter* | 0.02 | 0.01 | 0.03 | 0.02 | 0.04 | 0.03 | 0.05 |
| *Parabacteroides* | 0.08 | 0.12 | 0.07 | 0.15 | 0.07 | 0.13 | 0.09 |
| *Parachlamydia* | 0.07 | 0.01 | 0.03 | 0.03 | 0.04 | 0.18 | 0.24 |
| *Paraprevotella* | 0.06 | 0.12 | 0.08 | 0.17 | 0.12 | 0.13 | 0.05 |
| *Parasutterella* | 0.14 | 0.09 | 0.16 | 0.19 | 0.16 | 0.08 | 0.10 |
| *Pasteuria* | 0.10 | 0.01 | 0.01 | 0.03 | 0.70 | 0.19 | 0.45 |
| *Phascolarctobacterium* | 1.07 | 1.04 | 1.15 | 1.32 | 0.87 | 0.50 | 0.65 |
| *Phaselicystis* | 0.25 | 0.06 | 0.22 | 0.13 | 0.65 | 0.29 | 0.48 |
| *Phenylobacterium* | 0.28 | 0.01 | 0.28 | 0.12 | 0.05 | 0.19 | 0.11 |
| *Phycisphaera* | 0.14 | 0.09 | 0.16 | 0.12 | 0.51 | 0.18 | 0.40 |
| *Planctomyces* | 0.06 | 0.02 | 0.05 | 0.05 | 0.15 | 0.10 | 0.11 |
| *Porphyromonas* | 0.52 | 0.54 | 0.55 | 0.65 | 0.45 | 0.16 | 0.30 |
| *Prevotella* | 1.87 | 2.16 | 1.61 | 2.85 | 1.73 | 2.11 | 1.58 |
| *Prolixibacter* | 0.57 | 0.58 | 0.52 | 0.74 | 0.48 | 0.39 | 0.38 |
| *Pseudoflavonifractor* | 0.10 | 0.06 | 0.10 | 0.13 | 0.09 | 0.14 | 0.06 |
| *Pseudolabrys* | 0.41 | 0.03 | 0.14 | 0.38 | 0.11 | 0.18 | 0.11 |
| *Pseudomonas* | 2.65 | 2.18 | 3.67 | 1.23 | 1.01 | 0.93 | 3.11 |
| *Psychrobacter* | 0.05 | 0.05 | 0.03 | 0.08 | 0.03 | 0.02 | 0.02 |
| *Rhizobium* | 0.06 | 0.01 | 0.07 | 0.02 | 0.07 | 0.19 | 0.31 |
| *Rhizomicrobium* | 1.05 | 0.33 | 1.14 | 1.16 | 0.09 | 0.24 | 0.22 |
| *Rhodococcus* | 0.02 | 0.02 | 0.01 | 0.00 | 0.01 | 0.02 | 0.01 |
| *Rhodopila* | 0.05 | 0.04 | 0.03 | 0.02 | 0.01 | 0.02 | 0.03 |
| *Rhodoplanes* | 1.32 | 0.37 | 0.76 | 0.82 | 0.67 | 1.58 | 0.50 |
| *Rikenella* | 0.01 | 0.02 | 0.01 | 0.01 | 0.01 | 0.02 | 0.01 |
| *Roseburia* | 0.03 | 0.04 | 0.05 | 0.06 | 0.07 | 0.14 | 0.08 |
| *Roseomonas* | 0.06 | 0.03 | 0.04 | 0.22 | 0.01 | 0.02 | 0.01 |
| *Rudaea* | 0.18 | 0.01 | 0.18 | 0.08 | 0.01 | 0.03 | 0.01 |
| *Ruminobacter* | 0.03 | 0.04 | 0.03 | 0.05 | 0.01 | 0.02 | 0.01 |
| *Ruminococcus* | 0.67 | 0.75 | 0.82 | 1.10 | 0.76 | 0.56 | 0.57 |
| *Saccharofermentans* | 0.02 | 0.01 | 0.01 | 0.04 | 0.04 | 0.14 | 0.04 |
| *Sarcina* | 0.01 | 0.03 | 0.03 | 0.08 | 0.03 | 0.13 | 0.02 |
| *Schwartzia* | 0.02 | 0.04 | 0.02 | 0.06 | 0.04 | 0.02 | 0.02 |
| *Serratia* | 0.41 | 0.13 | 0.40 | 0.29 | 0.61 | 1.19 | 1.40 |
| *Simkania* | 0.24 | 0.01 | 0.03 | 0.07 | 0.06 | 0.08 | 0.09 |
| *Singulisphaera* | 0.17 | 0.19 | 0.17 | 0.05 | 0.22 | 0.08 | 0.11 |
| *Skermanella* | 0.25 | 2.30 | 1.51 | 0.35 | 0.09 | 0.03 | 1.02 |
| *Solibacillus* | 0.57 | 0.76 | 0.47 | 0.99 | 0.44 | 0.27 | 0.39 |
| *Solirubrobacter* | 0.05 | 0.10 | 0.16 | 0.02 | 0.23 | 0.06 | 0.08 |
| *Spartobacteria_genera_incertae_sedis* | 6.20 | 0.02 | 0.16 | 0.53 | 3.83 | 1.64 | 1.23 |
| *Sphingomonas* | 1.04 | 0.17 | 0.55 | 0.42 | 2.31 | 3.59 | 2.79 |
| *Sporobacter* | 0.75 | 0.86 | 0.94 | 1.14 | 0.76 | 1.29 | 0.70 |
| *Stella* | 0.54 | 0.72 | 0.34 | 0.35 | 0.18 | 0.19 | 0.18 |
| *Steroidobacter* | 0.17 | 0.19 | 0.34 | 0.20 | 1.64 | 1.82 | 1.08 |
| *Streptococcus* | 0.16 | 0.08 | 0.31 | 0.13 | 0.26 | 0.77 | 0.46 |
| *Streptomyces* | 0.20 | 0.04 | 0.03 | 0.01 | 0.09 | 0.06 | 0.09 |
| *Streptophyta* | 0.01 | 0.02 | 0.01 | 0.00 | 0.05 | 0.03 | 0.02 |
| *Subdivision3_genera_incertae_sedis* | 2.29 | 0.36 | 1.13 | 1.66 | 3.94 | 2.51 | 2.41 |
| *Subdoligranulum* | 0.06 | 0.04 | 0.06 | 0.06 | 0.01 | 0.03 | 0.03 |
| *Succinivibrio* | 13.97 | 20.56 | 19.55 | 22.92 | 11.43 | 10.18 | 9.30 |
| *Sutterella* | 0.05 | 0.05 | 0.04 | 0.06 | 0.06 | 0.06 | 0.05 |
| *Tannerella* | 0.12 | 0.19 | 0.12 | 0.21 | 0.14 | 0.13 | 0.10 |
| *Terrimonas* | 0.14 | 0.03 | 0.04 | 0.24 | 1.21 | 1.45 | 0.57 |
| *Thermoleophilum* | 2.34 | 1.98 | 1.92 | 0.56 | 1.11 | 0.21 | 0.96 |
| *Thermomonospora* | 0.03 | 0.16 | 0.28 | 0.02 | 0.01 | 0.05 | 0.01 |
| *Thermosporothrix* | 0.29 | 0.28 | 0.04 | 0.43 | 0.01 | 0.05 | 0.11 |
| TM7*_genera_incertae_sedis* | 2.17 | 3.72 | 2.41 | 3.72 | 2.54 | 1.95 | 0.99 |
| *Treponema* | 0.03 | 0.04 | 0.05 | 0.06 | 0.05 | 0.13 | 0.09 |
| *Trichococcus* | 0.23 | 0.32 | 0.24 | 0.46 | 0.24 | 0.13 | 0.21 |
| *Vampirovibrio* | 0.21 | 0.13 | 0.16 | 0.22 | 0.18 | 0.16 | 0.17 |
| *Variovorax* | 0.01 | 0.01 | 0.01 | 0.00 | 0.06 | 0.08 | 0.10 |
| *Veillonella* | 0.17 | 0.03 | 0.33 | 0.10 | 0.10 | 0.19 | 0.23 |
| *Verrucomicrobium* | 0.01 | 0.01 | 0.01 | 0.00 | 0.09 | 0.21 | 0.09 |
| *Zavarzinella* | 0.10 | 0.02 | 0.07 | 0.03 | 0.31 | 0.06 | 0.11 |
| **Abundance of total shared genera (% of all classified genera/sequences) in each sample** | **92.85** | **95.49** | **93.53** | **95.23** | **85.12** | **81.81** | **83.47** |
