# Supplementary material for: The rhizospheric microbial community structure and diversity of deciduous and evergreen forests in Taihu Lake area, China
Source: PLoS One. 2017 Apr 5;12(4):e0174411. doi: 10.1371/journal.pone.0174411 (PMC5381875; doi:10.1371/journal.pone.0174411)
Supplement: S6 Table — The percentages (% of total good-quality sequences) of 12 dominant genera are shown in the last line. (DOCX) [file pone.0174411.s009.docx]

**S6 Table.** **Relative abundances (% of total good-quality sequences) of twelve dominant genera (>1% of the classified sequences) in at least 5 soil samples.** The percentages (% of total good-quality sequences) of 12 dominant genera are shown in the last line.

| **Genus** | **GH** | **HB** | **KC** | **ZS** | **YX** | **ZT** | **ZW** |
| --- | --- | --- | --- | --- | --- | --- | --- |
| *Succinivibrio* | 20.56 | 19.55 | 22.92 | 13.97 | 10.18 | 9.30 | 11.43 |
| Gp2 | 19.17 | 11.12 | 6.07 | 3.81 | 0.34 | 5.70 | 0.09 |
| Gp1 | 8.77 | 8.96 | 8.79 | 8.84 | 0.82 | 2.34 | 0.10 |
| TM7*_genera_incertae_sedis* | 3.72 | 2.41 | 3.72 | 2.17 | 1.95 | 0.99 | 2.54 |
| *Barnesiella* | 3.57 | 2.94 | 3.11 | 2.17 | 1.75 | 1.42 | 1.55 |
| *Acinetobacter* | 2.51 | 1.89 | 4.27 | 1.78 | 1.30 | 1.37 | 1.65 |
| *Pseudomonas* | 2.18 | 3.67 | 1.23 | 2.65 | 0.93 | 3.11 | 1.01 |
| *Prevotella* | 2.16 | 1.61 | 2.85 | 1.87 | 2.11 | 1.58 | 1.73 |
| *Lachnospiracea_incertae_sedis* | 1.36 | 1.26 | 1.95 | 1.47 | 1.48 | 0.89 | 1.14 |
| *Gemmatimonas* | 1.16 | 0.78 | 0.96 | 4.62 | 1.54 | 3.11 | 2.25 |
| Gp3 | 1.07 | 2.12 | 4.58 | 3.12 | 1.82 | 1.25 | 1.26 |
| *Subdivision3_genera_incertae_sedis* | 0.36 | 1.13 | 1.66 | 2.29 | 2.51 | 2.41 | 3.94 |
| **The percentage (% of total good-quality sequences) of 12 dominant genera** | **66.58** | **57.45** | **62.11** | **48.74** | **26.72** | **33.46** | **28.70** |
