# Supplementary material for: The rhizospheric microbial community structure and diversity of deciduous and evergreen forests in Taihu Lake area, China
Source: PLoS One. 2017 Apr 5;12(4):e0174411. doi: 10.1371/journal.pone.0174411 (PMC5381875; doi:10.1371/journal.pone.0174411)
Supplement: S7 Table — (DOCX) [file pone.0174411.s010.docx]

**S7 Table.** **Relative abundances (>1% of total classified sequences) of dominant genera in each evergreen tree soil sample.**

| **Phylum** | **Genus** | **GH** | **HB** | **KC** | **ZS** |
| --- | --- | --- | --- | --- | --- |
| *Proteobacteria* | *Succinivibrio* | 20.56 | 19.55 | 22.92 | 13.97 |
| *Proteobacteria* | *Acinetobacter* | 2.51 | 1.89 | 4.27 | 1.78 |
| *Proteobacteria* | *Pseudomonas* | 2.18 | 3.67 | 1.23 | 2.65 |
| *Acidobacteria* | Gp3 | 1.07 | 2.12 | 4.58 | 3.12 |
| *Acidobacteria* | Gp2 | 19.17 | 11.12 | 6.07 | 3.81 |
| *Acidobacteria* | Gp1 | 8.77 | 8.96 | 8.79 | 8.84 |
| *Bacteroidetes* | *Barnesiella* | 3.57 | 2.94 | 3.11 | 2.17 |
| *Bacteroidetes* | *Prevotella* | 2.16 | 1.61 | 2.85 | 1.87 |
| *Firmicutes* | *Lachnospiracea_incertae_sedis* | 1.36 | 1.26 | 1.95 | 1.47 |
| *Firmicutes* | *Phascolarctobacterium* | 1.04 | 1.15 | 1.32 | 1.07 |
| TM7 | TM7*_genera_incertae_sedis* | 3.72 | 2.41 | 3.72 | 2.17 |
| **The percentage of dominant genera in evergreen tree rhizospheric soil samples** | | **66.10** | **56.68** | **60.81** | **42.89** |
