# Supplementary material for: The rhizospheric microbial community structure and diversity of deciduous and evergreen forests in Taihu Lake area, China
Source: PLoS One. 2017 Apr 5;12(4):e0174411. doi: 10.1371/journal.pone.0174411 (PMC5381875; doi:10.1371/journal.pone.0174411)
Supplement: S8 Table — (DOCX) [file pone.0174411.s011.docx]

**S8 Table.** **Relative abundances (>1% of total classified sequences) of dominant genera in each deciduous tree soil sample.**

| **Phylum** | **Genus** | **YX** | **ZT** | **ZW** |
| --- | --- | --- | --- | --- |
| *Proteobacteria* | *Succinivibrio* | 10.18 | 9.30 | 11.43 |
| *Proteobacteria* | *Acinetobacter* | 1.30 | 1.37 | 1.65 |
| *Proteobacteria* | *Steroidobacter* | 1.82 | 1.08 | 1.64 |
| *Proteobacteria* | *Sphingomonas* | 3.59 | 2.79 | 2.31 |
| *Proteobacteria* | *Dongia* | 2.97 | 1.34 | 2.17 |
| *Acidobacteria* | Gp3 | 1.82 | 1.25 | 1.26 |
| *Acidobacteria* | Gp4 | 2.77 | 3.06 | 4.22 |
| *Acidobacteria* | Gp5 | 1.58 | 1.42 | 1.23 |
| *Acidobacteria* | Gp6 | 7.77 | 7.85 | 10.48 |
| *Bacteroidetes* | *Barnesiella* | 1.75 | 1.42 | 1.55 |
| *Bacteroidetes* | *Prevotella* | 2.11 | 1.58 | 1.73 |
| *Verrucomicrobia* | *Subdivision3_genera_incertae_sedis* | 2.51 | 2.41 | 3.94 |
| *Verrucomicrobia* | *Spartobacteria_genera_incertae_sedis* | 1.64 | 1.23 | 3.83 |
| *Nitrospira* | *Nitrospira* | 1.24 | 1.64 | 1.41 |
| *Gemmatimonadetes* | *Gemmatimonas* | 1.54 | 3.11 | 2.25 |
| **The percentage of dominant genera in deciduous tree rhizospheric soil samples** | | **44.57** | **40.84** | **51.12** |
